# Medicinal Plants and Lead Phytomolecules as Immunomodulators: An Updated Review

**DOI:** 10.1155/bmri/6998034

**Published:** 2026-02-02

**Authors:** Gurdeep Singh, Vikas Sharma, Blessing Nnenna Udeh

**Affiliations:** ^1^ School of Pharmaceutical Sciences, Lovely Professional University, Phagwara, Punjab, India, lpu.in

**Keywords:** immunomodulatory, medicinal plants, multiple sclerosis, rheumatoid arthritis

## Abstract

All therapeutic interventions aimed at modulating the immune response to pathogens, self‐antigens, carcinogens, or xenogeneic antigens are referred to as immunomodulation, which either prevents hyperactivation or restores the appropriate response of the immune system. Since antiquity, medicinal plants have been used as a source of immune‐boosting medicines with fewer side effects. These medicinal plants, for example, *Curcuma longa*, *Camellia sinensis*, *Artemisia annua*, and *Andrographis paniculata*, have been used to treat immune system disorders such as multiple sclerosis, psoriasis, lupus, and organ transplantation. Herein, we review the currently accessible medicinal plants, their phytoconstituents, and underlying mechanisms of immunomodulation.

## 1. Introduction

The immune system is the body′s primary defense mechanism, protecting against pathogens while maintaining tolerance to self. It is broadly divided into innate immunity, which provides immediate nonspecific protection through cytokines, granulocytes, macrophages, mast cells, and basophils, and adaptive immunity, which generates specific responses via B and T lymphocytes and establishes long‐term memory. Both arms act in coordination to ensure effective host defense [1].

When this balance is disrupted, it can result in autoimmune diseases, allergies, hypersensitivity, immunodeficiency, or transplant rejection. The rising prevalence of such disorders highlights the urgent need for effective immunomodulators. Synthetic agents are available but often limited by poor bioavailability, stability issues, and adverse effects [2].

In contrast, medicinal plants and their phytoconstituents have long been valued for their immune‐regulating properties with fewer side effects. They can stimulate suppressed immune functions in immunodeficiency or downregulate hyperactivation in autoimmunity and transplant reactions. Their broad mechanisms involve modulation of cytokines, transcription factors, and immune cell activity. After destroying the antigen, T cells and B cells are in charge of controlling and stopping immune reactions to avoid triggering additional immunological responses.

This review provides an updated synthesis of medicinal plants with immunomodulatory potential, their phytochemical constituents, and underlying mechanisms of action, offering insight into their therapeutic promise as safer and more effective alternatives to synthetic drugs [3].

## 2. Classification of Immunomodulators

Immunomodulators are biological or synthetic agents that activate, suppress, or modify immune system components. Based on their activity, they are classified as immunostimulants, immunosuppressants, and immunoadjuvants.

### 2.1. Immunostimulants

Immunostimulants enhance the body′s defense mechanisms against infections, allergies, autoimmunity, or cancer by stimulating B or T cells and other immune components. They are often nonspecific and require repeated administration to maintain therapeutic efficacy [4].

### 2.2. Immunosuppressants

Immunosuppressants downregulate or inhibit immune activity, controlling autoimmune diseases, hypersensitivity reactions, graft‐versus‐host disease, and organ transplant rejection. By lowering immune reactivity, they improve graft survival and are also termed antirejection agents [5].

### 2.3. Immunoadjuvants

Immunoadjuvants are used in vaccines to boost antigen‐specific responses. They enhance phagocytosis, provide slow antigen release, and stimulate cytokine production, helping to differentiate between protective and destructive immune responses. Despite their potential, only a few plant‐derived adjuvants (e.g., saponins, polysaccharides, and glycyrrhizin) have shown promise for clinical application [6].

Table 1 summarizes major phytochemicals categorized as immunostimulants, immunosuppressants, or immunoadjuvants.

**Table 1:** Some major phytochemicals categorized as immunosuppressants, immunostimulants, or immunoadjuvants.

**Immunomodulatory properties**	**Phytochemical**	**Source**
Immunoadjuvants	Andrographolide	*Andrographis paniculata*
	Ganoderic acid T	*Ganoderma lucidum*
	Glycyrrhizin	*Glycyrrhiza glabra*
	Tanshinones	*Salvia miltiorrhiza*

Immunomodulators	Allicin	*Allium sativum*
	Baicalin	*Scutellaria baicalensis*
	Berberine	*Berveris vulgaris*
	Boswellic acids	*Boswellia serrata*
	Curcumin	*Curcuma longa*
	Quercetin	*Moringa oleifera*
	Resveratrol	*Polygonum cuspidatum*
	Rutin	*Sambucus javanica*
	Withaferin A	*Withania somnifera*

Immunostimulants	Astragalus	*Astragalus membranaceus*
	Echinacoside	*Echinacea purpurea*
	Epigallocatechin gallate	*Camellia sinensis*
	Ginseng	*Panax ginseng*
	Ginsenosides	*Panax ginseng*

Immunosuppressants	Triptolide	*Tripterygium wilfordii*

## 3. Merits and Demerits of Synthetic Immunomodulators

There are many different types of immunomodulators available in the market, including natural, synthetic, and recombinant substances. Synthetic immunomodulators offer several merits, including precise targeting of immune pathways, consistent quality, and scalability in production, which make them valuable in treating autoimmune diseases, cancers, and infections. They can be structurally optimized for potency, stability, and reduced side effects compared with natural compounds. However, they also present demerits such as the risk of immune overactivation or suppression, which may lead to serious side effects like bone necrosis, osteopenia, nephrotoxicity, neurotoxicity, pancytopenia, hemorrhagic cytosis, increased susceptibility to infections, or autoimmune reactions. Additionally, high development costs, potential long‐term toxicity, and the need for thorough clinical evaluation are significant challenges associated with their use, necessitating the search for more potent and secure agents with immunomodulatory activity. Thus, the drawbacks of synthetic immunomodulators highlight the need for safer and more effective plant‐derived alternatives with better tolerability and sustainable efficacy [7].

## 4. Method Section and Search Strategy

**Figure 1:**
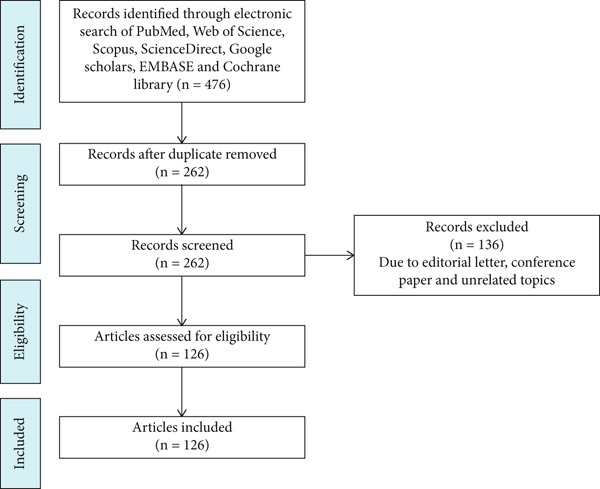
Study selection process.

The most current literature was specifically deepened by screening manuscripts from 2010 to 2022 from major scientific databases like PubMed, Web of Science, Scopus, ScienceDirect, Google Scholar, EMBASE, and Cochrane Library using keywords that is, phytochemicals, immunomodulators, immunosuppressants, rheumatoid arthritis, and plant‐based immunomodulators (Figure 1).

## 5. Plant‐Derived Immunomodulators

**Figure 2:**
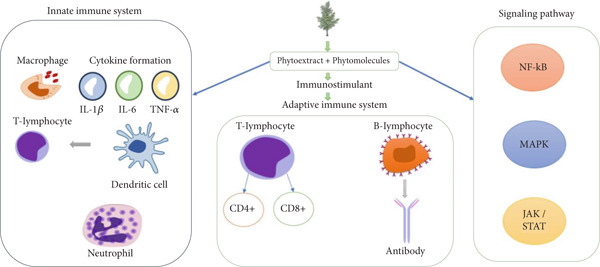
Mechanism of medicinal plants in immunomodulation.

Before modern medicine, there were herbal or traditional medicines, which were used to cure illnesses in several different systems, including Ayurveda (India), Western Chinese, Kampo (Japan), and Unani (South Asia). The therapeutic effects of traditional remedies are currently being examined through studies on plant species. Ayurveda is one of the earliest systems of conventional medicine, which uses ethnopharmacological techniques to treat illnesses like cancer, rheumatoid arthritis, stress, and immune disorders. There is one of the eight main disciplines of Ayurveda, called Rasayana, that is concerned with improving immunity and the resistance of the body. Immunomodulatory properties have been claimed for some medicinal herbs, such as Rasayana, which consists of a number of plants that promote physical and mental health, and promote the body defense system, and longevity [8]. *Withania somnifera, Tinospora cordifolia*, *Glycyrrhiza glabra, Phyllanthus emblica* are among the medicinal herbs used in Rasayana that are said to still have immunomodulatory properties [9]. These phytochemicals regulate the immune response through enhanced stimulation or repressive host defenses (Figure 2). Below is a list of plants that possess immunomodulatory activities (Table 2).

**Table 2:** Immunomodulatory effects of various fractions of plants.

**Botanical name; common names; family**	**Part used/solvent used for extraction**	**Dose; route of administration**	**Immunomodulatory mechanism**	**References**
*Aloe vera*; Indian aloe (Aspholdelaceae)	Leaves/ethanol	100–400 mg/kg; oral	• Increases phagocytosis • Promotes superoxide levels	[10]
*Andrographis paniculata*; kalmegh (Acanthaceae)	Leaves, root/aqueous ethanol	400 mg/kg; oral	• Increases IL‐2 levels • Inhibits of NO production	[11]
*Artemisia annua;* wormwood (Asteraceae)	Entire herb/ethanol	15 mg/kg; intraperitoneal	• Reduces TLR2 levels • Alters TLR4 expression • Inhibits TLR4, MyD88, and NF‐*κ*B expression	[12]
*Azadirachta indica;* neem**/**margo satree (Meliaceae)	Leaves, aqueous/ethanol bark	100 mg/kg; oral	• Increases IgM and IgG production • Inhibits NO production	[13]
*Asparagus racemosus*; satawar (Asparagacae)	Roots/aqueous	100 mg/kg; oral	• Increases the production of leukocytosis • Enhances the phagocytic activity	[14]
*Bidens pilosa;* beggar**-**ticks (Asteraceae)	Flowers, leaves/aqueous	5–10 mg/kg; intraperitoneal	• Enhances the cytokine/WBCs levels • Increases IFN‐*α* promoter activity • Enhances IFN‐*γ* activity	[15]
*Camellia sinensis*; tea (Theaceae)	Leaves/aqueous	1.5–25 *μ*g/mL; intraperitoneal	• Enhances neopterin production	[16]
*Cannabis sativa*; brahmi (Cannabaceae)	Leaves/ethanol	75 mg/kg; oral	• Decreases TNF‐*α* and INF‐*γ* levels	[17]
*Centella asiatica;* (Apiaceae)	Entire plant/ethanol	100 mg/kg; oral	• Increases WBC count • Inhibits production of IL‐2 and TNF‐*α*	[18]
*Cistanche deserticola*; cistanche (Orobanchaceae)	Entire herb/aqueous Ethanol	1–5 g/kg; oral	• Upregulates cytotoxicity • Reduces oxidative stress	[19]
*Curcuma longa;* turmeric (Zingiberaceae)	Rhizome/aqueous	20 mg/kg; oral	• Decreases ROS, hepatic SOD, and GSH levels • Upregulates TBRAS, TNF‐*α*, and IL‐6 mRNA	[20]
*Echinacea angustifolia*; coneflower (Asteraceae)	Flowers/aqueous ethanol	50 mg/kg; oral	• Increases T cell proliferation	[21]
*Euphorbia hirta;* asthma weed (Euphorbiaceae)	Entire herb/aqueous	25 mg/kg; intraperitoneal	• Inhibits NO production	[22]
*Glycyrrhiza glabra* licorice (Leguminosae)	Rhizomes/aqueous ethanol	500 mg/kg; oral	• Enhances immune activities. • Stimulates immune cells	[23]
*Mangifera indica*; mango tree (Anacardiaceae)	Bark/ethanol	100–300 mg/kg; oral	• Increase in humoral antibody (HA) titer • Enhancement of IgG1 and IgG2b production	[24]
*Matricaria chamomilla;* chamomile (Asteraceae)	Flowers/ethanol	20 mg/animal	• Activation of immune cells • Inhibits the production of NO	[25]
*Moringa oleferia;* drumstick tree (Moringaceae)	Leaves/aqueousethanol	250–1000 mg/kg; oral	• Decreases TGF‐*β* and IFN‐*γ* levels • Downregulates NF‐*κ*B expression	[26]
*Ocimum sanctum*; holybasil**/**tulsi (Lamiaceae)	Leaf extract/aqueous Ethanol	250 mg/kg; oral	• Reduces leucocyte migration • Reduces production of histamine	[27]
*Panax ginseng;* ninjin (Araliaceae)	Flower/ethanol	25–100 mg/kg; oral	• Upregulation of NO and iNOS • Activates TNF‐*α* and IFN‐*γ* production	[28]
*Phyllanthus emblica;* Indian gooseberry (Punicaceae)	Fruit bark/ethanol	12–50 mg/kg; oral	• Modulates imunosuppressive effects • Restoration of IL‐2 and IFN‐*γ* production.	[29]
*Picrorhiza kurroa;* picrorhiza (Plantaginaceae)	Root/aqueous	12–50 mg/kg; oral	• Enhances levels of cytokines (IFN‐*γ* and IL‐4) • Enhances lymphocytes′ proliferation	[30]
*Tinospora cordifolia*; giloy (Menispermaceae)	Entire plant/Methanol	5–100 *μ*g/mL	• Increases white blood cell count • Enhances macrophage activation	[31]
*Withania somnifera*; winter cherry (Solanaceae)	Root/aqueous	150–300 mg/kg; oral	• Increases total WBC count • Increases antibody titer • Increases phagocytic activity of macrophages	[32]

### 5.1. *Artemisia annua* Wild.

**Figure 3:**
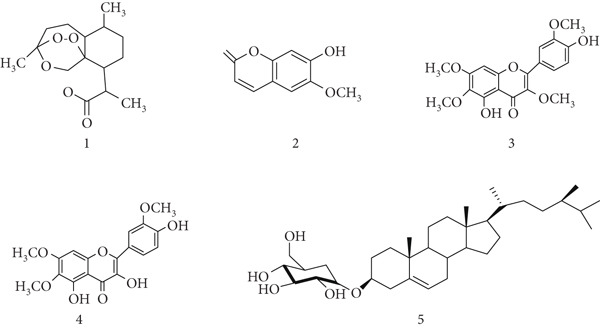
Chemical structures of constituents of *Artemisia annua.*

*A. annua* (Asteraceae) is the primary source of artemisinin, a sesquiterpene lactone with potent antimalarial activity. Artemisinin and its derivatives, including artemether, artesunate, and dihydroartemisinin, are effective against both chloroquine‐sensitive and resistant *Plasmodium falciparum* strains. [33, 34]. *A. annua* contains some bioactive chemical constituents (Figure 3), mainly (1) artemisinin , (2) scopoletin, (3) chrysosplenetin , (4) eupatin , and (5) 3‐O‐*β*‐D‐glucopyranoside of sitosterol.

Beyond antimalarial use, extracts of *A. annua* exhibit immunomodulatory properties. In a study, ethanolic extracts of *A. annua* suppress immune function by inhibiting Con A‐ and LPS‐stimulated splenocyte proliferation in a dose‐dependent manner, leading to reduced cellular and humoral responses in mice [35]. Islamuddin M. et al. reported that *A. annua* extract in BALB/c mice reduced IgG1, increased IgG2a, and enhanced delayed‐type hypersensitivity (DTH) responses, marked by elevated IFN‐*γ* and reduced IL‐4/IL‐10, indicating a shift toward Th1 immunity [36].

In *Acanthamoeba*‐infected mice, *A. annua* water extracts downregulated TLR2 and altered TLR4 expression, indicating anti‐inflammatory effects [37, 38]. Additionally, polysaccharide fractions (AAPs) enhanced IL‐6 and TNF production, with AAP‐1 showing strong immunostimulatory activity and low toxicity [39].

### 5.2. *Azadirachta indica* A. Juss

**Figure 4:**
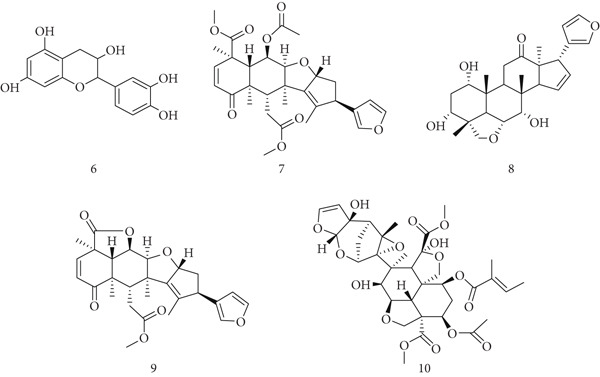
Chemical structures of constituents of *Azadirachta indica.*

*A. indica* (Meliaceae), commonly known as neem, is widely distributed across Africa, America, and India and has long been used in traditional medicine. Its bioactive constituents include (6) epicatechin, (7) nimbin, (8) nimbidin, (9) nimbolide, and (10) azadirachtin (Figure 4). Neem exhibits broad biological activities, including antibacterial, antifungal, anti‐inflammatory, and antipyretic effects [40]. Polysaccharides and limonoids from bark, leaves, and seed oil demonstrate antitumor activity, reducing tumor size and showing efficacy against lymphocytic leukemia. Ethanolic leaf extracts decreased papilloma incidence and tumor burden in Swiss albino mice, whereas neem seed oil inhibited breast tumor growth, reducing tumor volume by ~50% in the MCF‐7 model. Neem also induces apoptosis in cancer cells (e.g., prostate PC‐3) in a dose‐dependent manner, with nimbolide identified as a potent mitochondrial apoptosis inducer [41]. Furthermore, purified neem leaf extract suppresses procancer inflammatory mediators including IL‐1, IL‐6, NF‐*κ*B, COX‐2, TNF‐*α*, and IFN‐*γ* [42]. Collectively, these studies highlight neem′s role in immune modulation and cancer prevention.

### 5.3. *Curcuma longa* L.

**Figure 5:**
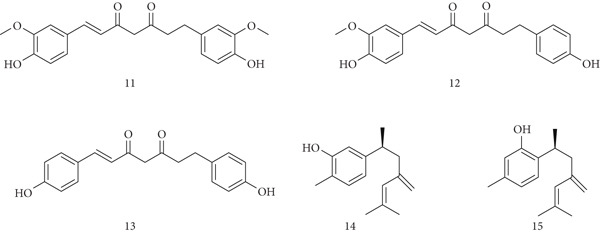
Chemical structures of constituents of *Curcuma longa.*

*C. longa* (turmeric), cultivated widely in India, China, and Southeast Asia, is used both as a food additive and traditional medicine. It exhibits anti‐inflammatory, anticoagulant, hepatoprotective, and immunostimulant properties [43]. The main bioactive compounds are curcuminoids and other bioactive compounds like (11) curcumin, (12) demethoxycurcumin, (13) bisdemethoxycurcumin, and (14) turmeronols A and (15) B (Figure 5). Curcumin acts through multiple pathways by modulating transcription factors, kinases, and cytokines, leading to tumor inhibition and apoptosis. It blocks NF‐*κ*B activation, reverses chemoresistance in pancreatic cancer, and reduces angiogenesis and tumor growth in animal models [44, 45]. Curcumin also prevents oxidative liver injury, demonstrating antioxidant and hepatoprotective activity [46].

At the immune level, curcumin regulates innate and adaptive responses by interacting with dendritic cells, macrophages, B and T cells, and modulating cytokines. It suppresses IL‐1, IL‐2, IL‐6, IL‐8, TNF‐*α*, IFN‐*γ*, MCP‐1, iNOS, and NO [47–49]. In murine *Klebsiella pneumonia* infection, direct curcumin delivery improved survival, reduced bacterial load, and lowered inflammatory mediators in lung tissue and blood [50]. Curcumin also enhances stem cell immunomodulatory properties, osteogenic and chondrogenic differentiation, while regulating PGE2–IDO signaling and glycolysis [51]. Both in vitro and in vivo studies confirm its dual role in boosting T lymphocyte‐mediated immunity and inducing apoptosis in cancer cells [52, 53].

### 5.4. *Picrorhiza kurroa* Royle Ex Benth

**Figure 6:**
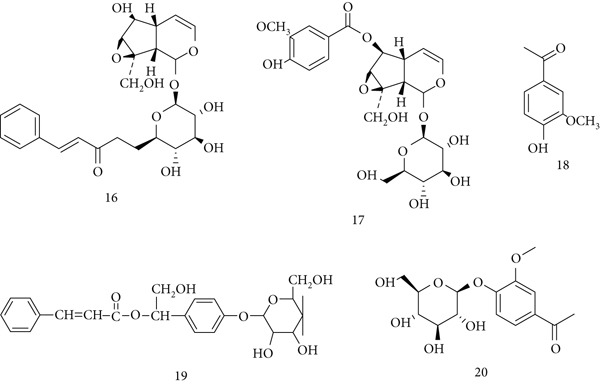
Chemical structures of constituents of *Picrorhiza kurroa.*

*P. kurroa* (Scrophulariaceae) has been traditionally used in Ayurveda for liver disorders, respiratory illnesses, and chronic fevers. Its pharmacological actions—antioxidant, anti‐inflammatory, hepatoprotective, and anticancer—are attributed to iridoid glycosides such as (16, 17) picrosides I–II, (18) apocynin, (19) kutkin, and (20) androsin (Figure 6) [54]. Ethanolic leaf extract (PKLE) enhanced cell‐mediated and humoral immunity in mice, inducing early and delayed hypersensitivity reactions, boosting phagocytosis, and improving reticuloendothelial activity [55]. Rhizome extract showed anti‐inflammatory effects, dose‐dependently inhibiting carrageenan‐induced paw edema and granuloma formation in rats [56]. In cancer models, *P. kurroa* extract reduced tumor incidence and mortality after 20‐methylcholanthrene exposure, suppressed ascites tumor growth, and inhibited proliferation in *Saccharomyces cerevisiae* mutants [57]. These findings confirm its role as an immunostimulant with anti‐inflammatory and anticancer potential.

### 5.5. *Moringa oleifera* L.

**Figure 7:**
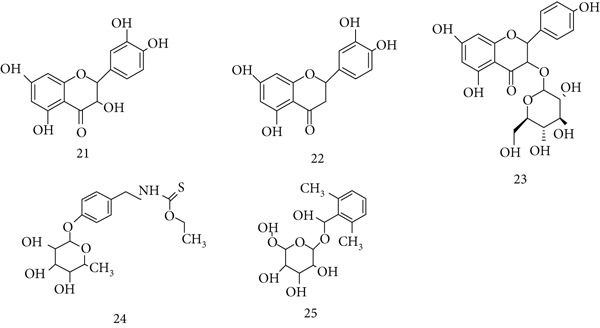
Chemical structures of constituents of *Moringa oleifera.*

*M. oleifera* (Moringaceae), known as the drumstick or horseradish tree, is widely valued for its nutritional and medicinal properties. Its bioactive constituents include (21) quercetin, (22) luteolin, (23) astragalin, (24) niazimicin, and (25) moringyn (Figure 7). Rich in amino acids, carotenoids, vitamins, and polyphenols, *M. oleifera* demonstrates broad pharmacological effects. Water leaf extract showed cardiomodulating activity in isolated frog hearts, likely due to alkaloids [58], whereas hydroethanolic extracts reduced blood pressure in L‐NAME‐induced hypertensive rats from 159.6 to 102.4 mmHg [59]. Fermented *M. oleifera* leaf extract enhanced resistance against *Salmonella typhi* infection in mice, proving more effective than nonfermented preparations [60]. Additionally, two polysaccharides (MOP‐1 and MOP‐2) activated macrophages, increasing ROS, NO, IL‐6, and iNOS production, confirming strong immunoregulatory activity [61, 62].

### 5.6. *Cannabis sativa* L.

**Figure 8:**
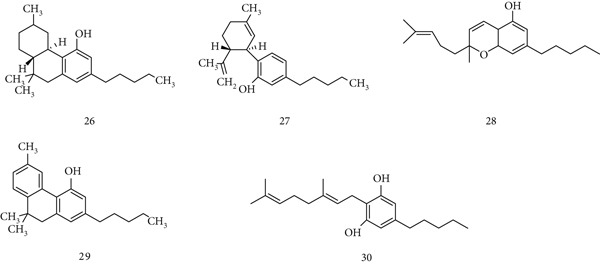
Chemical structures of constituents of *Cannabis sativa.*

*C. sativa* (Cannabinaceae), also known as Indian hemp, has been used historically for food and medicine but is now more commonly associated with recreational abuse [63]. Its main phytocannabinoids include (26) *Δ* ^9^‐tetrahydrocannabinol (THC), (27) cannabidiol (CBD), (28) cannabichromene (CBC), (29) cannabinol (CBN), and (30) cannabigerol (CBG) (Figure 8). Cannabis and its constituents show therapeutic potential in conditions such as chronic pain, cancer, epilepsy, spasticity, neurodegenerative, and psychiatric disorders [64]. Cannabinoids exert immunomodulatory effects by inducing apoptosis, suppressing cell proliferation, reducing pro‐inflammatory cytokines/chemokines, enhancing anti‐inflammatory cytokines, and promoting regulatory T cells [65]. They also act on cancer‐related pathways (PKB, AMPK, mTOR, HIF‐1, and PPAR), leading to apoptosis, cell cycle arrest, and inhibition of tumor proliferation [66]. However, CBD has been linked to dose‐dependent hepatotoxicity, whereas THC may interact with immunosuppressants (e.g., tacrolimus) and CNS depressants, necessitating monitoring during therapeutic use.

### 5.7. *Mangifera indica* L.

**Figure 9:**
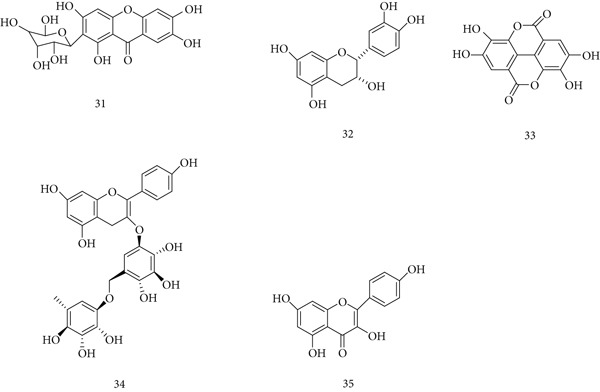
Chemical structures of constituents of *Mangifera indica.*

*M. indica* (Anacardiaceae), commonly known as mango (aam), is widely used in traditional medicine across Africa and South Asia. Different parts of the plant—including bark, leaves, fruit peel, and seed kernel—exhibit antibacterial, antiviral, anti‐inflammatory, antimalarial, and immunostimulant properties [67]. Major bioactive compounds include (31) mangiferin, (32) epicatechin, (33) ellagic acid, (34) kaempferol‐3‐O‐rutinoside, and (35) quercetin (Figure 9). An alcoholic stem bark extract (2.6% mangiferin) enhanced humoral antibody titers and DTH in mice, indicating immunostimulant activity [68]. Similarly, a methanolic extract (160 mg/kg) boosted both innate and adaptive immunity in SRBC‐challenged mice by increasing WBC, SI, HA, and DTH responses [69]. Mango peel ethanol extract also suppressed IgE production in human myeloma cell lines, suggesting a role for mangiferin in allergy regulation [70].

### 5.8. *W. somnifera* (L.) Dunal

**Figure 10:**
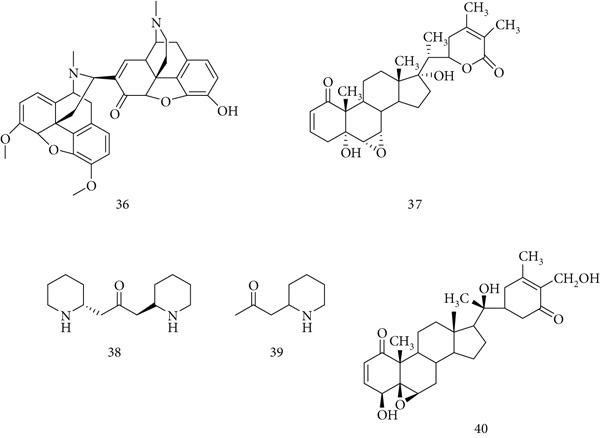
Chemical structures of constituents of *Withania somnifera.*

*W. somnifera* is a key Ayurvedic herb with broad pharmacological effects, including antioxidant, adaptogenic, memory‐enhancing, antiparkinson, antivenom, anti‐inflammatory, and anticancer properties. Its bioactive constituents include (36) somniferin, (37) withanone, (38) anaferine, (39) isopelletierine, and (40) withanolides (Figure 10). Other components include steroidal lactones (ergostane derivatives), alkaloids (ashwagandha, cuscohygrine, anahygrine, and tropines), acylated saponins (sitoindosides VII–VIII), withaniol, acylsteryl glucosides, sugars, and hentriacontane [71].

In BALB/c mice, root extract (20 mg/dose) significantly enhanced immune responses, increasing WBC count, bone marrow density, and *α*‐esterase‐positive cells. Co‐administration with SRBC antigen elevated plaque‐forming cells and circulating antibodies, while enhancing macrophage phagocytosis and reducing DTH, confirming immunostimulatory potential [72]. *W. somnifera* glycoproteins also showed antivenom activity, inhibiting hyaluronidase from cobra (*Naja naja*) and viper (*Daboia russelii*) venoms [73]. Additionally, Taranjeet et al. reported that withania extract mitigated immune dysregulation caused by acute sleep deprivation by upregulating NF‐*κ*B, TNF‐*α*, and IL‐6 [74].

### 5.9. *Aloe vera* L.

**Figure 11:**
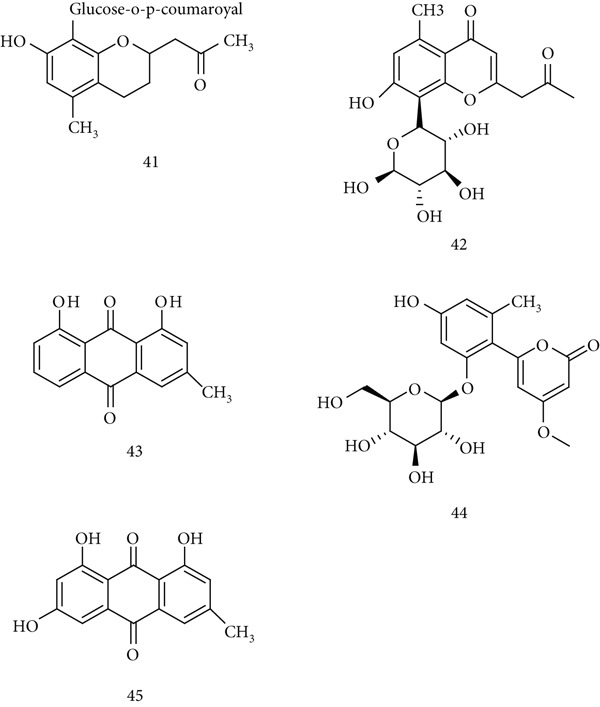
Chemical structures of constituents of *Aloe vera.*

*A. vera* (Asphodelaceae) has been used worldwide for centuries for medicinal and cosmetic purposes. It grows mainly in arid regions of Africa, Asia, Europe, and the Americas and exhibits wound‐healing, anti‐inflammatory, immunomodulatory, antiviral, antibacterial, and anticancer effects. Its key bioactive compounds include (41) aloe emodin, (42) aloesin, (43) aloin, (44) aloenin, and (45) emodin (Figure 11).

Studies demonstrate that *A. vera* regulates immune responses primarily through macrophage activation. Extracts improved the viability of inflamed murine macrophages infected with *Candida albicans*, with several fractions (R100, R50, R30, and R10) significantly enhancing cell survival [75]. Acemannan, a major polysaccharide, increased NO and IL‐6 production in peritoneal macrophages, further supporting immunostimulatory action [76]. In vivo, *A. vera* extract enhanced both humoral and cell‐mediated immunity in pigeons challenged with pigeon paramyxovirus type 1, mediated via activation of IFN genes, NF‐*κ*B proteins, and IL‐8 secretion [77].

### 5.10. *Asparagus racemosus* Willd.

**Figure 12:**
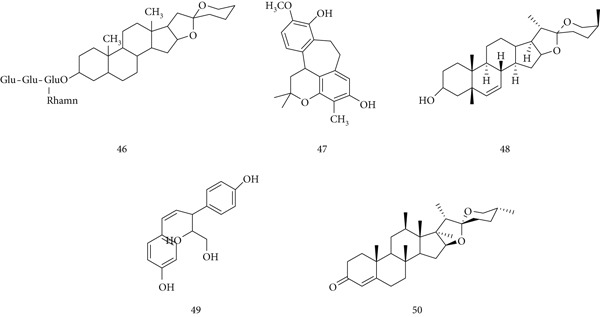
Chemical structures of constituents of *Asparagus racemosus.*

*A. racemosus* (Liliaceae), known as shatavari or satawar, grows at low elevations in India, and its dried roots are widely used in traditional medicine. Pharmacological effects include ulcer healing, galactagogue activity, and tonic properties. Major bioactive compounds are (46) shatavarins, (47) racemosol A, (48) sarsasapogenin, (49) isoagatharesinol, and (50) asparacosin A (Figure 12).

Animal studies demonstrate immune‐enhancing activity. Oral root decoctions produced leukocytosis, neutrophilia, and enhanced macrophage and polymorph phagocytosis. Alcoholic root extracts promoted mammary gland development and increased milk secretion in rats, attributed to prolactin and corticoid release [78]. Immunological studies in SRBC‐sensitized rats showed that aqueous root extracts increased antibody titers, lymphocyte proliferation, and CD3^+^ and CD4^+^/CD8^+^ cell percentages. This was accompanied by upregulation of both Th1 (IL‐2 and IFN‐*γ*) and Th2 (IL‐4) cytokines, indicating dual Th1/Th2 adjuvant activity [79]. Additionally, shatavarins from *A. racemosus* cell cultures stimulated human peripheral blood lymphocytes, enhancing IgG secretion and IL‐12 production while suppressing IL‐6 [80].

### 5.11. *T. cordifolia* (Willd.) Miers

**Figure 13:**
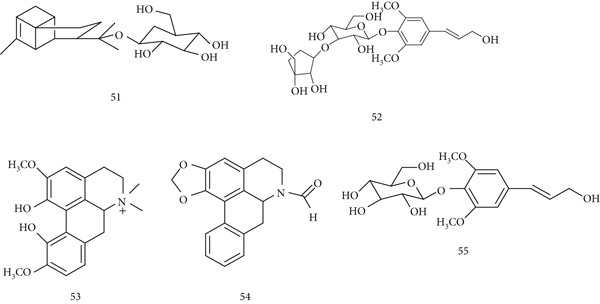
Chemical structures of constituents of *T. cordifolia.*

*T. cordifolia* (Menispermaceae) is widely used in traditional medicine and is reported to have hepatoprotective, immunomodulatory, anti‐inflammatory, antioxidant, antistress, and anticancer activities. Its active constituents include (51) tinocordiside, (52) cordifolioside A, (53) magnoflorine, (54) N‐formylannonain, and (55) syringin (Figure 13).

In BALB/c mice, T. cordifolia extract enhanced *antibody production* against ovalbumin, with 5–7‐fold increases in IgG and 3–5‐fold increases in IgA, while also elevating the splenic index, confirming both immunogenic and adjuvant activity [81]. Oral aqueous extracts provided antioxidant and hepatoprotective effects in a paracetamol‐induced liver toxicity model [82]. Additionally, ethanolic stem extract inhibited *Toxoplasma gondii* invasion and intracellular replication, showing efficacy comparable to clindamycin [83].

### 5.12. *Panax ginseng* Meyer

**Figure 14:**
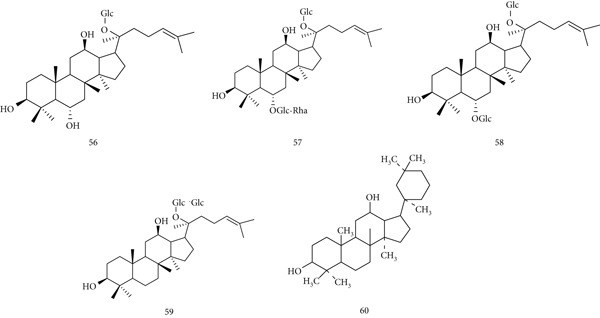
Chemical structures of constituents of *Panax ginseng.*

*P. ginseng* (Araliaceae) is one of the most widely known medicinal herbs, used for centuries as a tonic and immune modulator. Roots, stems, and leaves are employed to support immunological homeostasis and enhance disease resistance. Major bioactive compounds include (56) Ginsenosides F1, (57) Ginsenosides Re, (58) Ginsenosides Rg1, (59) Ginsenosides Rb1, and (60) panaxadiol (Figure 14).

Both in vivo and in vitro studies confirm immunomodulatory effects. *P. ginseng* protects against *Listeria monocytogenes* infection and restores NK cell function in cyclophosphamide‐treated mice, while stimulating TNF and IFN‐*γ* production in spleen cells and macrophages via TLR‐4 [84]. Ginseng and ginsenosides also show anti‐inflammatory activity, reducing proinflammatory cytokines through hypothalamic–pituitary–adrenal (HPA) axis regulation, while enhancing NK cell activity and phagocytosis [85]. Ginsenoside Rd promotes regulatory T cell development by upregulating Foxp3 and increasing TGF‐*β*1, IL‐10, and IL‐35, suggesting applications in transplantation and autoimmune disease [86]. Ginseng polysaccharides further enhance macrophage activation, stimulating IL‐1, IL‐6, IL‐12, TNF, and NO production [87].

### 5.13. *G. glabra* L.

**Figure 15:**
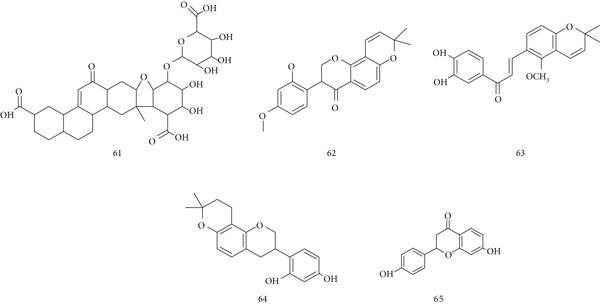
Chemical structures of constituents of *G. glabra.*

*G. glabra* (Fabaceae) is well known for its ethnopharmacological value, containing phytocompounds such as isoflavones, glabrin A and B, 18‐glycyrrhetinic acid, and glycyrrhizin with antibacterial, anti‐inflammatory, antiviral, antioxidant, and antidiabetic activities [88]. Key active constituents include (61) glycyrrhizic acid, (62) glabroisoflavonone A, (63) glycyglabrone, (64) glabriin, and (65) liquiritigenin (Figure 15).

An aqueous root extract demonstrated immunostimulant properties, reducing mortality in septic mice, enhancing *Escherichia coli* phagocytosis in the carbon clearance test, and increasing DTH and hemagglutination antibody titers [89]. Glycyrrhizic and glycyrrhetinic acids also modulated immunity by upregulating iNOS and transcription factors such as NF‐*κ*B, STAT3, and STAT6 [90]. However, excessive glycyrrhizin intake may cause pseudohyperaldosteronism, hypokalemia, hypertension, and fluid retention through inhibition of 11*β*‐HSD2, indirectly affecting liver function. In psoriasis models, glycyrrhizin suppressed IL‐17A and IFN‐*γ* expression in vivo and inhibited IL‐17A‐HaCaT cell proliferation in vitro by upregulating SIRT1 and reducing STAT3 signaling, improving skin pathology [91].

### 5.14. *P. emblica* L.

**Figure 16:**
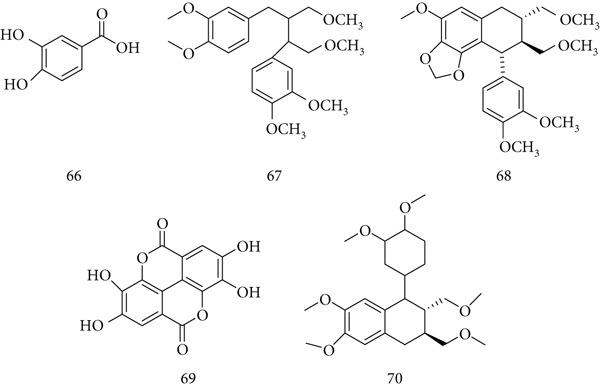
Chemical structures of constituents of *P. emblica.*

*P. emblica* Linn. (Euphorbiaceae), also known as Indian gooseberry or amla, has long been used in traditional medicine. Its fruits and seeds are rich in flavonoids, glycosides, proanthocyanidins, gallic acid, emblicanin, ellagic acid, and Vitamin C, which contribute to antioxidant, anticancer, hepatoprotective, and immunomodulatory effects. Major active constituents include (66) gallic acid, (67) phyllanthin, (68) hypophyllanthin, (69) ellagic acid, and (70) phyltetralin (Figure 16) [92].

Experimental studies demonstrate anticancer and immunoprotective properties. Amla extract reduced benzopyrene‐ and heavy metal–induced genotoxicity in mice and decreased skin tumor volume by 60% at 100 mg/kg. Polyphenol‐rich fractions (60–250 mg/kg) inhibited N‐nitrosodiethylamine–induced hepatocellular carcinoma (HepG2) by 80%–100%, though effects varied with different carcinogens [93]. Quercetin, a key flavonoid, has consistently reduced tumor growth in animal models. Amla fruit extract also protected thymocytes from arsenic‐induced oxidative stress by reducing ROS, lipid peroxidation, and caspase‐3 activity, while restoring antioxidant enzyme activity and mitochondrial potential [94]. In alcohol‐exposed rats, *P. emblica* extract decreased NO, protein carbonyls, and lipid peroxides, while enhancing NADH dehydrogenase, succinate dehydrogenase, and cytochrome c oxidase activity [95].

### 5.15. *Andrographis paniculata* Wall. Ex Nees

**Figure 17:**
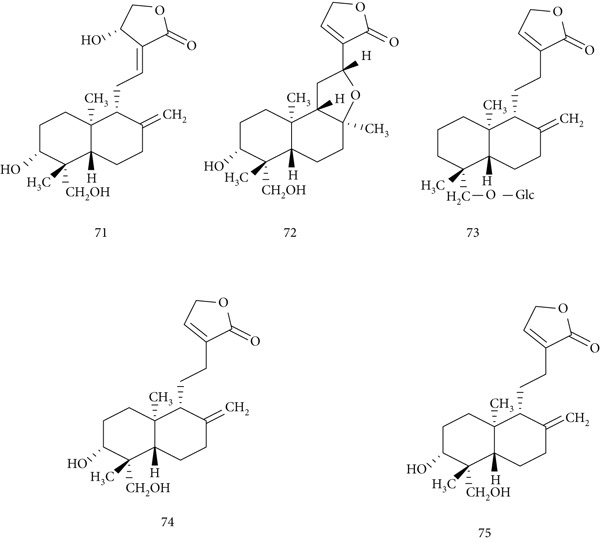
Chemical structures of constituents of *A. paniculata.*

*A. paniculata* Nees (Acanthaceae), known as Kalmegh, is widely used in Indian and Chinese traditional medicine. Its major bioactive constituents, (71) andrographolide, (72) isoandrographolide, (73) neoandrographolide, (74) 14‐deoxy‐11, 12‐didehydroandrographolide, and (75) 14‐deoxyandrographolide (Figure 17), exhibit anticancer, anti‐inflammatory, antidiabetic, antimalarial, and antiviral activities [96].

Recent studies suggest that *A. paniculata* may complement modern therapies in HIV/AIDS by interfering with viral signal transduction, enzyme function, and replication [97]. Its immunostimulant activity operates via both antigen‐specific antibody production and nonspecific activation of macrophages to clear pathogens [98]. Rajanna et al. reported that extracts significantly increased T cells, T helper cells, and cytokines including IFN‐*γ*, IL‐2, and IL‐4, confirming strong immunomodulatory potential [99]. Additional studies highlight its role in augmenting host antiviral responses [100].

### 5.16. *Ocimum sanctum* L.

**Figure 18:**
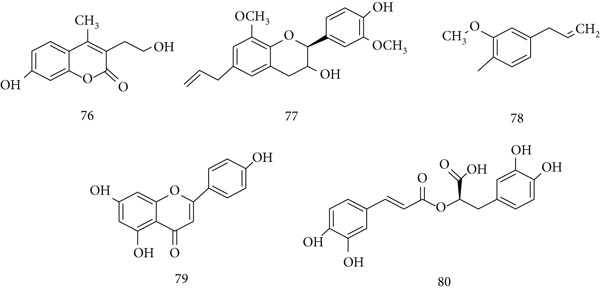
Chemical structures of constituents of *O. sanctum.*

*O. sanctum* (Tulsi) has been used in traditional medicine worldwide, with various parts (leaves, stem, root, seeds, and flowers) applied against skin disorders, malaria, diarrhea, and dysentery. It exhibits antifertility, anticancer, antidiabetic, antifungal, antibacterial, cardioprotective, and analgesic effects. Key constituents include (76) ocimarin, (77) tulsinol A, (78) eugenol, (79) apigenin, and (80) rosmarinic acid (Figure 18) [101, 102].

In in vivo studies, ethanolic extracts lowered blood glucose, glycosylated hemoglobin, and urea while increasing glycogen, hemoglobin, and protein in streptozotocin‐induced diabetic rats, suggesting stimulatory effects on insulin secretion. Extracts also mimicked insulin activity in normal rats, with hypoglycemic potency comparable to tolbutamide [103]. Preclinical data indicate that Tulsi and its phytochemicals (eugenol, rosmarinic acid, apigenin, sitosterol, orientin, and vicenin) protect against chemical‐induced organ damage and radiation injury by enhancing antioxidant defenses, modulating gene expression, and inducing apoptosis. These findings support its chemopreventive and radioprotective roles [104]. Immunomodulatory effects were observed in a BALB/c mice model of visceral leishmaniasis, where *O. sanctum* increased DTH response, shifted humoral immunity toward Th1, and restored liver function [105]. In dairy cows with mastitis, Tulsi therapy eradicated intramammary infections, reduced inflammation, improved milk quality, and enhanced neutrophil phagocytic activity, demonstrating strong immunotherapeutic potential [106].

### 5.17. *Echinacea angustifolia* (DC.) A. Helle

**Figure 19:**
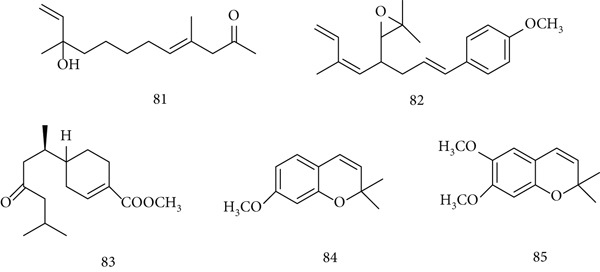
Chemical structures of constituents of *E. angustifolia.*

*E. angustifolia* (Asteraceae), commonly used to treat cold symptoms, is valued for its immunostimulatory and anti‐inflammatory properties. Traditionally applied in chemotherapy and chemoprevention of respiratory infections, it also shows antioxidant, anticancer, and antiviral effects. Major constituents include (81) echinolone, (82) juvocimine‐2, (83) juvabione, (84) precocine I, and (85) precocine II (Figure 19).

In in vivo studies, root dry powder or alcohol extracts increased splenic T cell proliferation and NK cell cytotoxicity. Alcohol extracts from three *Echinacea* species enhanced NK cell activity, PFC response to sRBC, B and T lymphocyte proliferation, and cytokine production in BALB/c mice, supporting both innate and adaptive immune activation [107]. Dendritic cells exposed to whole plant, stem and leaf, flower, and root extracts for 24 h showed reduced HLA‐DR and CD32 expression, with the strongest inhibition from whole plant and stem‐leaf extracts, suggesting suppression of dendritic cell maturation [108]. In another study, *E. angustifolia* extract (100 *μ*g/mL) activated murine bone marrow‐derived macrophages (M1), upregulating CD80, CD86, MHCII, and CCR7, and increasing IL‐1, IL‐6, IL‐12p70, TNF, NO, phagocytosis, and bactericidal activity [109].

### 5.18. *Bidens pilosa* Linn.

**Figure 20:**
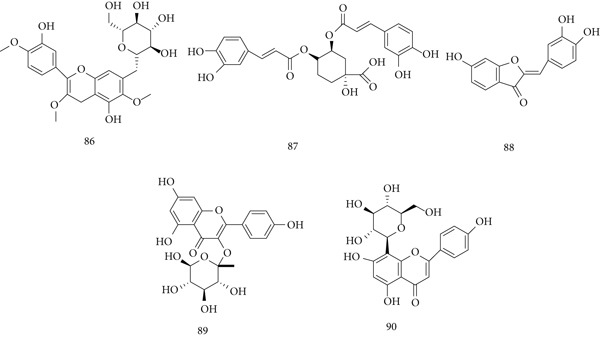
Chemical structures of constituents of *B. pilosa.*

*Bidens pilosa* Linn. (beggar‐ticks), originally from South America, is now widespread globally. The whole plant, including aerial parts and roots, is used in traditional medicine as powders, tinctures, macerations, or decoctions. Major constituents are (86) centaurine, (87) 3, 4‐di‐o‐caffeoylquinic acid, (88) sulfuretin, (89) astragalin, and (90) vitexin (Figure 20). It exhibits pharmacological effects against inflammation, immune disorders, infections, malignancies, metabolic syndrome, and wounds.

Extracts of *B. pilosa* showed cytotoxic activity in in vitro assays. Using MTT, extracts inhibited cervical carcinoma (HeLa) and HepG2 cells, with IC_50_ values of 14.80 and 13.50 *μ*g/mL after 48 h [110]. Chang et al. reported that centaurein, a flavonoid from *B. pilosa*, modulates IFN‐*γ* expression in Jurkat cells and regulates NFAT and NF‐*κ*B activity, highlighting its immunomodulatory potential [111].

### 5.19. *Camellia sinensis* (L.) Kuntze

**Figure 21:**
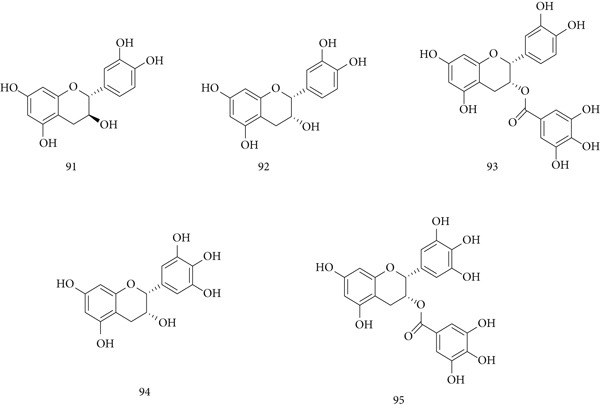
Chemical structures of constituents of *C. sinensis.*

*C. sinensis* (Theaceae), commonly known as green tea, is cultivated in India, China, and many regions worldwide. It has multiple pharmacological benefits attributed to constituents such as (91) catechin, (92) epicatechin, (93) epicatechin‐3‐gallate, (94) epigallocatechin, and (95) epigallocatechin‐3‐gallate (EGCG) (Figure 21).

In in vivo studies, tea extract (250–500 mg/kg, p.o.) administered to albino mice for up to 45 days enhanced immune responses [112]. In immunocompromised Wistar rats infected with *C. albicans*, green tea extract showed stronger immunomodulatory effects than EGC or EGCG, increasing IL‐8, IL‐17A, and HBD‐2 expression [113]. Noncatechin flavonoids from seeds improved TNF‐*α*‐impaired insulin signaling and glucose uptake, showing antimetabolic and anti‐inflammatory effects [114]. Additionally, triterpenoid saponins from tea leaves selectively inhibited human ovarian cancer cells by inducing apoptosis via the extrinsic pathway and suppressing angiogenesis [115].

### 5.20. *Matricaria chamomilla* L.

**Figure 22:**
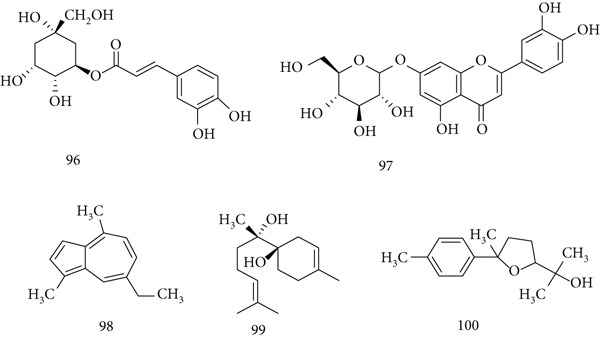
Chemical structures of constituents of *M. chamomilla.*

*M. chamomilla* (Asteraceae), native to Europe and Asia, has demonstrated anxiolytic, antimutagenic, cholesterol‐lowering, wound‐healing, and antidiabetic effects in animal studies. Its main constituents include (96) chlorogenic acid, (97) luteolin‐7‐O‐glucoside, (98) chamazulene, (99) *α*‐bisabolol, and (100) bisabolol oxide B (Figure 22).

In BALB/c mice, chamomile extract increased bone marrow cellularity and spleen weight (*p* < 0.01). In cyclophosphamide‐immunosuppressed mice, pretreatment with extract restored resistance to lethal *C. albicans* infection, largely dependent on granulocytes (*p* < 0.01). These findings confirm its immunomodulatory potential, suggesting value in preventing opportunistic infections and as supportive therapy in oncology [25].

### 5.21. *Centella asiatica* Linn.

**Figure 23:**
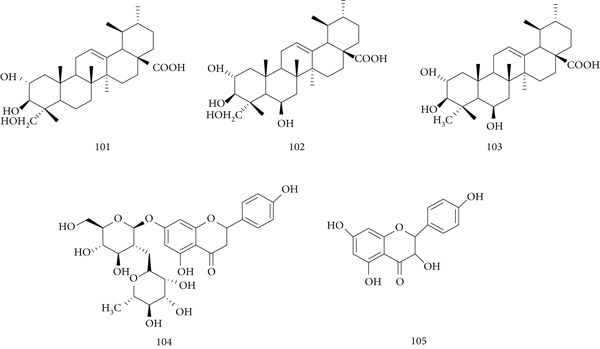
Chemical structures of constituents of C*. asiatica.*

*Centella asiatica* (Umbellifere/Apiceae), a perennial creeper common in India, was historically used in Western medicine for leprosy treatment. Its major active compounds are triterpenoid saponins such as asiaticosides. Key constituents include (101) asiatic acid, (102) madecassic acid, (103) medasiatic acid, (104) naringin, and (105) kaempferol **(**Figure 23 **).**

In vitro studies showed dose‐dependent immunomodulatory activity of PKLE (25–100 mg/mL), enhancing neutrophil migration and phagocytic index compared with controls. In vivo assays demonstrated similar effects: in cyclophosphamide‐induced myeloid suppression, methanolic extract (500 mg/kg BW) increased WBC counts in Swiss albino mice. Carbon clearance tests further confirmed dose‐dependent enhancement of phagocytic activity. However, no significant increase in antibody production was observed [116].

### 5.22. Cistanche deserticola Y. C. Ma

**Figure 24:**
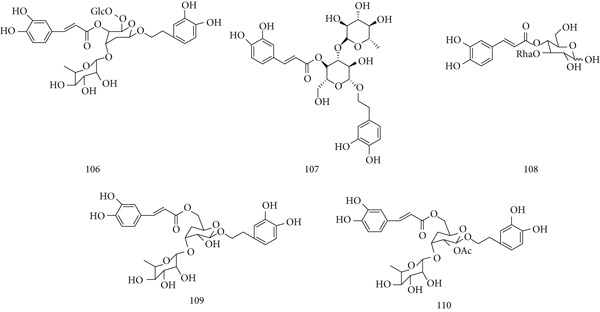
Chemical structures of constituents of *C. deserticola.*

*C. deserticola* Y.C. Ma, native to arid regions of China and India, has long been used in traditional Chinese medicine as a tonic. Its pharmacological activities include immunomodulatory, antioxidant, hepatoprotective, and antiviral effects. Major constituents are (106) echinacoside, (107) acteoside, (108) cistanoside F, (109) isoacteoside, and (110) tubuloside B **(**Figure 24 **)**, with polysaccharides identified as the most active components.

In in vivo studies, aqueous extract of *C. deserticola* (AECD) enhanced immune responses to inactivated FMD vaccine by increasing IgG, IL‐4, lymphocyte proliferation, and balanced Th1/Th2 activity. AECD also promoted CD4^+^, CD8^+^, CD44^+^ T cell activation, IFN‐*γ* production, CTL response, and neutralizing antibodies, while reducing Treg frequency and upregulating CD80, CD40, MHC‐II, and CD86 on dendritic cells, suggesting potential as a polysaccharide‐based vaccine adjuvant [117]. Water‐extractable polysaccharides (WPCD) similarly increased T and B cell proliferation, IFN‐*γ*, IL‐4, IgG1, and IgG2a titers, while lowering Tregs and enhancing CD40/CD80 expression on splenic DCs [118]. Another study confirmed aqueous extracts of cultivated *C. deserticola* activate dendritic cells via the TLR4–NF‐*κ*B pathway, regulating cytokine secretion and maturation [119].

### 5.23. *Euphorbia hirta* Linn.

**Figure 25:**
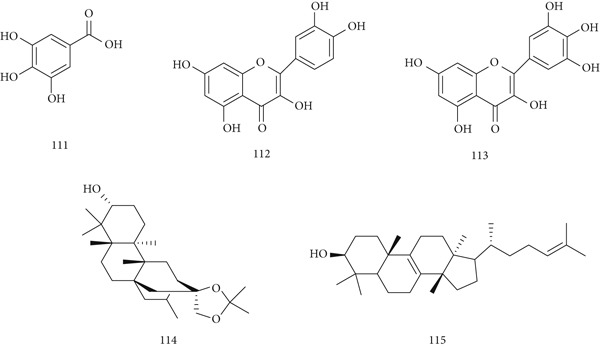
Chemical structures of constituents of *E. hirta.*

*Euphorbia hirta* Bunge (EHB), a traditional Chinese medicine, is used for expectoration, cough, asthma, detoxification, and itching. Modern studies confirm its anticancer, antibacterial, and antioxidant properties. Major constituents include (111) gallic acid, (112) quercetin, (113) myricetin, (114) euphorneroid D, and (115) euphol (Figure 25). In vitro experiments evaluated the effects of methanolic *E. hirta* extract on MCF‐7 breast cancer cells. At concentrations of 1.96–250 *μ*g/mL, the extract showed cytotoxicity with IC_50_ of 25.26 *μ*g/mL after 24 h. Microscopic analysis revealed characteristic apoptotic morphology, whereas flow cytometry, annexin V staining, and assays for DNA fragmentation, caspase activation, and ROS generation further confirmed apoptosis. Bioassay‐guided fractionation subsequently identified the specific cytotoxic fractions of *E. hirta* extract responsible for these effects [120].

## 6. Effect of Immunomodulators on Some Autoimmune Disorders

Curcumin has been shown to suppress IL‐12 production in macrophages and to inhibit IL‐12 signaling in T cells—effects that led to the increased production of IL‐4 and to reciprocally decreased interferon‐*γ* production by T cells. Furthermore, curcumin‐treated T cells inhibit IL‐12–induced T cell proliferation and Th1 differentiation [121].

### 6.1. Multiple Sclerosis

It is a serious health issue that deteriorates over time. The immune system of the body attacks the fatty myelin sheaths that surround the nerve cells (neurons) in the central nervous system in this illness. Medicinal plants like *C. longa, P. ginseng, C. sinensis*, and many therapeutic effects of *C. sativa* have been found in MS patients. The most significant and widely utilized plant in the treatment of MS is *C. sativa*. THC is the main substance found in *C. sativa* . THC acts as a partial agonist to CB1 and CB2 receptors by binding to cannabinoid receptors (CBR) in the central nervous system. THC used orally can lessen the severity of several MS signs and symptoms, including stiffness, rigidity, and tremor, as well as improve walking abilities and bladder control. It has been proven that smoking marijuana can help MS sufferers with their stiffness, pain, tremors, and emotional dysfunction [121].

### 6.2. Lupus

An autoimmune disease known as lupus causes the body′s immune system to become overactive and target healthy, normal tissue over an extended period of time. Inflammation, swelling, and harm to the joints, skin, kidneys, blood, heart, and lungs are among the symptoms. Clinical trials and animal studies suggest that artemisinin may have benefits for SLE. The benefits of artemisinin therapy include alleviating symptoms, lowering antibody and proteinuria levels, minimizing kidney damage, and reducing prednisone dosage. Artemisinin may exert its effects via modulating T cell subsets, preventing B cell activation and the release of inflammatory cytokines, and inhibiting anti‐inflammatory and immunomodulatory processes, according to animal studies. Artemisinin and its derivatives are a promising possible new medicinal medicine that may pose a threat to the present lupus treatment [122].

### 6.3. Psoriasis

Psoriasis is a chronic autoimmune circumstance that causes the speedy build‐up of skin cells and pores. In research that was done with the triterpenes glycyrrhizin and glycyrrhetinic acid of liquorice on pores and skin, an in vivo mouse model of imiquimod (IMQ)–induced psoriasis‐like inflammation (IPI) was used to study the impact of GL on psoriasis. The effects of GL therapy on IPI‐affected animals were found to reduce inflammation severity and postpone the emergence of IPI lesions in mice. Investigating the molecular processes underlying the GL‐mediated effects of IPI, it was discovered that GL reduced ICAM‐1 expression in the lesions of mice treated with IMQ. Consequently, this study demonstrated a novel ICAM‐1 expression‐modulating role for GL in psoriasis by regulating NF‐*κ*B and ERK/p38 MAPK pathways in keratinocytes [123].

### 6.4. Rheumatoid Arthritis

Rheumatoid arthritis is an autoimmune and inflammatory disorder, which means that your immune system erroneously attacks healthy cells in your body, causing inflammation in the affected areas of your body. The joints are the main areas of rheumatoid arthritis′s influence, and it commonly affects several joints at once. The most typical joints afflicted by RA are the hands, wrists, and knees. Juices of *M. oleifera* and decoctions of *O. sanctum* are found to be anti‐arthritic. By using a formaldehyde‐induced arthritis model, *M. oleifera* plant extracts were studied in Wistar rats at dose levels of 150, 300, and 600 mg/kg. These extracts prevented arthritis‐induced anemia in rats and significantly reduced paw inflammation in a dose‐dependent manner. The study′s findings indicated that all *M. oleifera* extracts were effective. Extracts showed significant antioxidant and anti‐arthritic potential in rats, and these effects were dose dependent [124].

## 7. Effect of Immunomodulators on Graft Rejection

Graft rejection occurs when the recipient′s immune system attacks transplanted tissue or organs due to recognition of foreign HLA antigens. Allografts (between genetically nonidentical individuals of the same species) are particularly prone to rejection, necessitating immunosuppressive therapy. *C. longa* (curcumin) has shown potential in transplant immunology. Curcumin inhibits cytokines, chemokines, and NF‐*κ*B, suppresses IL‐12 production in macrophages, and interferes with IL‐12 signaling in T cells. This leads to increased IL‐4 and reduced IFN‐*γ* production, along with inhibition of Th1 differentiation and T cell proliferation, suggesting curcumin as a possible adjunct in managing graft rejection [125].

## 8. Clinical Relevance and Pharmacokinetics of Key Phytochemicals

To bridge the gap between traditional medicinal plant usage and evidence‐based clinical practice, it is essential to evaluate not only the immunomodulatory efficacy of phytochemicals but also their clinical relevance, formulations in use, and pharmacokinetic profiles. The following summary (Table 3) highlights key plant‐derived compounds with demonstrated immunomodulatory potential.

**Table 3:** Clinical relevance and pharmacokinetics of key phytochemicals.

**Phytochemical (plant source)**	**Clinical trials/human studies**	**Formulations in use**	**Pharmacokinetics highlights**
Curcumin (*Curcuma longa*)	RCTs in rheumatoid arthritis, IBD, COVID‐19; ↓TNF‐*α*, IL‐1*β*, IL‐6	Capsules, liposomal curcumin, curcumin–piperine, nanoformulations	Poor bioavailability; rapid metabolism; enhanced by piperine/nanoforms
Resveratrol (*Vitis vinifera*)	Human studies in SLE, T2DM, obesity; modulates NF‐*κ*B, IL‐6	Tablets, resveratrol‐enriched foods, liposomal formulations	Rapid metabolism; low oral bioavailability; peak plasma ~1–2 h
Andrographolide (*Andrographis paniculata*)	Clinical use in respiratory infections, RA; ↑Th1, ↓IL‐6	KalmCold, standardized extract capsules	Plasma t½ ~2.7 h; low water solubility; metabolized hepatically
Boswellic acids (*Boswellia serrata*)	Clinical studies in OA, asthma, colitis; ↓5‐LOX, TNF‐*α*	Shallaki, Phytosome, Boswellia extract tablets	Poor oral bioavailability; enhanced by lecithin‐based carriers
Withaferin A (*Withania somnifera*)	Trials in anxiety, cancer, aging; immunomodulation via NF‐*κ*B, HSPs	Ashwagandha root extract capsules	Oral t½ ~6–7 h; CYP450 metabolism; improved in withanolide‐rich extracts
EGCG (*Camellia sinensis*)	Trials in cancer, obesity, viral infections; ↓TNF‐*α*, T cell modulation	Green tea extract, EGCG capsules	Oral absorption ~0.1%–0.3%; peak levels in 1.5 h; glucuronidation common
Glycyrrhizin (*Glycyrrhiza glabra*)	Used in SARS, hepatitis; trials show TLR4 inhibition, ↓IL‐6	Deglycyrrhizinated licorice, syrups, lozenges	Prodrug; hydrolyzed to glycyrrhetic acid; enterohepatic circulation
Allicin (*Allium sativum*)	Human studies in immunity, infection prevention; ↓CRP, ↑NK activity	Garlic capsules, aged garlic extract	Unstable in vivo; rapidly metabolized to allyl sulfur compounds
Berberine (*Berberis aristata*)	Clinical trials in T2DM, dyslipidemia, inflammation; ↓TNF‐*α*, ↑AMPK	Berberine HCl capsules, sustained‐release tablets	Low oral bioavailability (~1%); hepatic metabolism; t½ ~4–6 h
Quercetin (various sources)	RCTs in allergic rhinitis, COVID‐19; inhibits mast cells, ↓IL‐8	Quercetin phytosome, capsules, food supplements	Poor solubility; improved with phytosomes/liposomes; rapid clearance
Cannabidiol (CBD) (*Cannabis sativa*)	Human trials in epilepsy, MS, anxiety; ↓IL‐1*β*, TNF‐*α*; ↑Tregs	Oral oil, soft gels, Epidiolex, nano‐CBD	Variable bioavailability; hepatic metabolism; t½ ~18–32 h
Baicalin (*Scutellaria baicalensis*)	Human data in viral infections, allergic diseases; inhibits COX‐2, NF‐*κ*B	Capsules, decoctions in TCM, baicalin phytosomes	Low GI absorption; metabolized to baicalein; half‐life ~9 h

## 9. Conclusion

Various medicinal plants have been used for the treatment of different autoimmune disorders and other diseases. From the preceding discussion, it should be clear that many medicinal plants have immunomodulatory properties. Some can stimulate the immune system and some would suppress the immune system. These medicinal plants have less or no toxic effect on humans or the experimental models. Modulation of immunological responses via a phytoextract′s stimulatory or suppressive activity may aid in the maintenance of a disease‐free state in healthy or unwell people. It is clear from the review that a number of medicinal plants and their bioactive compounds have immunomodulatory action, but insufficient data prevent their use in clinical practice; hence, further research is needed to investigate them for clinical practice. Due to their great efficacy, cheap cost, and low toxicity, immunomodulatory drugs should become more important in the research of herbal medicine in the future.

## Conflicts of Interest

The authors declare no conflicts of interest.

## Funding

No funding was received for this manuscript.

## Data Availability

No underlying data was collected or produced in this study.

## References

[1] Nicholson L. B. , The Immune System, Essays in Biochemistry. (2016) 60, no. 3, 275–301, 10.1042/EBC20160017, 2-s2.0-85013428035. 27784777 PMC5091071

[2] Vesely M. D. , Kershaw M. H. , Schreiber R. D. , and Smyth M. J. , Natural Innate and Adaptive Immunity to Cancer, Annual Review of Immunology. (2011) 29, no. 1, 235–271, 10.1146/annurev-immunol-031210-101324, 2-s2.0-79953059789. 21219185

[3] Sethi J. and Singh J. , Role of Medicinal Plants as Immunostimulants in Health and Disease, Annals of Medicinal Chemistry Research. (2015) 1, no. 2.

[4] Shahbazi S. and Bolhassani A. , Immunostimulants: Types and Functions, Journal of Medical Microbiology and Infectious Diseases. (2016) 4, no. 3, 45–51.

[5] Wagner H. , Search for Plant Derived Natural Products With Immunostimulatory Activity: Recent Advances, Pure and Applied Chemistry. (1990) 62, no. 7, 1217–1222, 10.1351/pac199062071217, 2-s2.0-84955804702.

[6] Agarwal S. S. and Singh V. K. , Immunomodulators: A Review of Studies on Indian Medicinal Plants and Synthetic Peptides, Proceedings of the Indian National Science Academy-Part B: Biological Sciences. (1999) 65, no. 3-4, 179–204.

[7] Jantan I. , Ahmad W. , and Bukhari S. N. , Plant-Derived Immunomodulators: An Insight on Their Preclinical Evaluation and Clinical Trials, Frontiers in Plant Science. (2015) 6, 10.3389/fpls.2015.00655, 2-s2.0-84940650582, 26379683. PMC4548092 26379683

[8] Venugopalan S. N. and Venkatasubramanian P. , Understanding the Concepts Rasayana in Ayurveda Biology, Journal of Natural Ayurvedic Medicine. (2025) 1, no. 2, 1–12, 10.23880/jonam-16000112.

[9] Thamizhselvam N. and Swamy G. K. , Medicinal Plants in Rasayana Drugs, Their Active Ingredients and Reported Biological Activities: An Overview, Journal of Natural Ayurvedic Medicine. (2020) 4, no. 1, 1–5, 10.23880/jonam-16000227.

[10] Im S. A. , Lee Y. R. , Lee Y. H. , Lee M. K. , Park Y. I. , Lee S. , Kim K. , and Lee C. K. , In Vivo Evidence of the Immunomodulatory Activity of Orally Administered *Aloe vera* Gel, Archives of Pharmacal Research. (2010) 33, no. 3, 451–456, 10.1007/s12272-010-0315-1, 2-s2.0-77953648548, 20361311. 20361311

[11] Yao H. , Zhu J. , Xie Y. , Zhao N. , Yao R. , Sun H. , and Han G. , Protective Effect of the Effective Part of *Andrographis paniculata* (Burm. f.) Nees on PM2. 5-Induced Lung Injury in Rats by Modulating the NF-*κ*B Pathway, Journal of Ethnopharmacology. (2021) 280, 114420, 10.1016/j.jep.2021.114420, 34271116. 34271116

[12] Yao W. , Wang F. , and Wang H. , Immunomodulation of Artemisinin and Its Derivatives, Science Bulletin. (2016) 61, no. 18, 1399–1406, 10.1007/s11434-016-1105-z, 2-s2.0-84974853357.

[13] Subapriya R. and Nagini S. , Medicinal Properties of Neem Leaves: A Review, Current Medicinal Chemistry, Anti-Cancer Agents. (2005) 5, no. 2, 149–156, 10.2174/1568011053174828, 2-s2.0-14844301650, 15777222. 15777222

[14] Gautam M. , Diwanay S. , Gairola S. , Shinde Y. , Patki P. , and Patwardhan B. , Immunoadjuvant Potential of *Asparagus racemosus* Aqueous Extract in Experimental System, Journal of Ethnopharmacology. (2004) 91, no. 2-3, 251–255, 10.1016/j.jep.2003.12.023, 2-s2.0-2342445213, 15120447. 15120447

[15] Ashafa A. O. T. and Afolayan A. J. , Screening the Root Extracts From *Biden pilosa* L. Var. radiata (Asteraceae) for Antimicrobial Potentials, Journal of Medicinal Plants and Research. (2009) 3, no. 8, 568–572.

[16] Zvetkova E. , Wirleitner B. , Tram N. T. , Schennach H. , and Fuchs D. , Aqueous Extracts of *Crinum latifolium* (L.) and *Camellia sinensis* How Immunomodulatory Properties in Human Peripheral Blood Mononuclear Cells, International Immunopharmacology. (2001) 1, no. 12, 2143–2150, 10.1016/S1567-5769(01)00140-0, 2-s2.0-0034780010, 11710543. 11710543

[17] Nichols J. M. , Kummari E. , Sherman J. , Sherman E. J. , Dhital S. , Gilfeather C. , Yray G. G. , Morgan T. , and Kaplan B. L. F. , CBD Suppression of EAE Is Correlated With Early Inhibition of Splenic IFN-*γ* + CD8+ T Cells and Modest Inhibition of Neuroinflammation, Journal of Neuroimmune Pharmacology. (2021) 16, no. 2, 346–362, 10.1007/s11481-020-09917-8. 32440886 PMC7679272

[18] Punturee K. , Wild C. P. , Kasinrerk W. , and Vinitketkumnuen U. , Immunomodulatory Activities of *Centella asiatica* and *Rhinacanthus nasutus* Extracts, Asian Pacific Journal of Cancer. Prevention. (2005) 6, no. 3, 396–400, 16236006. 16236006

[19] Liu B. , Shi J. , Li Z. , Zhang C. , Liu P. , Yao W. , and Jia T. , Study on Neuroendocrine‐Immune Function of *Cistanche deserticola* and Its Rice Wine Steaming Products in Glucocorticoid‐Induced Rat Model, Evidence Based Complementary Alternative Medicine. (2020) 2020, no. 1, 5321976, 10.1155/2020/5321976, 33505484. 33505484 PMC7811494

[20] Uchio R. , Higashi Y. , Kohama Y. , Kawasaki K. , Hirao T. , Muroyama K. , and Murosaki S. , A Hot Water Extract of Turmeric (*Curcuma longa*) Suppresses Acute Ethanol-Induced Liver Injury in Mice by Inhibiting Hepatic Oxidative Stress and Inflammatory Cytokine Production, Journal of Nutritional Science. (2017) 6, 1–9, 10.1017/jns.2016.43, 2-s2.0-85009365856. PMC5465857 28620478

[21] El-Sherbiny E. M. , Osman H. F. , and Taha M. S. , Effectiveness of *Echinacea purpurea* Extract on Immune Deficiency Induced by Azathioprine in Male Albino Rats, Bioscience Journal. (2021) 37, no. e37029, 1981–3163, 10.14393/BJ-v37n0a2021-51270.

[22] Ramesh K. V. and Padmavathi K. , Assessment of Immunomodulatory Activity of *Euphorbia hirta* L, Indian Journal of Pharmaceutical Sciences. (2010) 72, no. 5, 621–625, 10.4103/0250-474X.78532, 2-s2.0-79955084012. 21694995 PMC3116308

[23] Ayeka P. A. , Bian Y. , Githaiga P. M. , and Zhao Y. , The Immunomodulatory Activities of Licorice Polysaccharides (*Glycyrrhiza uralensis* Fisch.) in CT 26 Tumor-Bearing Mice, BMC Complementary and Alternative Medicine. (2017) 17, no. 1, 10.1186/s12906-017-2030-7, 2-s2.0-85038107640, 29246138. PMC5732493 29246138

[24] Naved T. , Siddiqui J. I. , Ansari S. H. , Ansari A. A. , and Mukhtar H. M. , Immunomodulatory Activity of *Mangifera indica* L. Fruits (cv Neelam), Journal of Natural Remedies. (2005) 5, no. 2, 137–140.

[25] Ghonime M. , Eldomany R. , Abdelaziz A. , and Soliman H. , Evaluation of Immunomodulatory Effect of Three Herbal Plants Growing in Egypt, Immunopharmacology and Immunotoxicology. (2011) 33, no. 1, 141–145, 10.3109/08923973.2010.487490, 2-s2.0-79551598298. 20507215

[26] Nfambi J. , Bbosa G. S. , Sembajwe L. F. , Gakunga J. , and Kasolo J. N. , Immunomodulatory Activity of Methanolic Leaf Extract of *Moringa oleifera* in Wistar Albino Rats, Journal of Basic and Clinical Physiology and Pharmacology. (2015) 26, no. 6, 603–611, 10.1515/jbcpp-2014-0104, 2-s2.0-84948408203, 26103628. 26103628 PMC4630119

[27] Goel A. , Singh D. K. , Kumar S. , and Bhatia A. K. , Immunomodulating Property of *Ocimum sanctum* by Regulating the IL-2 Production and Its mRNA Expression Using Rat’s Splenocytes, Asian Pacific Journal of Tropical Medicine. (2010) 3, no. 1, 8–12, 10.1016/S1995-7645(10)60021-1, 2-s2.0-77950671498.

[28] Cui L. , Chen L. , Yang G. , Li Y. , Qiao Z. , Liu Y. , Meng Y. , Zhou Y. , and Sun L. , Structural Characterization and Immunomodulatory Activity of a Heterogalactan From *Panax ginseng* Flowers, Food Research International. (2021) 140, 109859, 10.1016/j.foodres.2020.109859, 33648177. 33648177

[29] Huabprasert S. , Kasetsinsombat K. , Kangsadalampai K. , Wongkajornsilp A. , Akarasereenont P. , Panich U. , and Laohapand T. , The *Phyllanthus emblica* L. Infusion Carries Immunostimulatory Activity in a Mouse Model, Journal of Medical Association of Thailand. (2012) 95, no. 2, S75–S82. 22574533

[30] Gupta A. , Khajuria A. , Singh J. , Bedi K. L. , Satti N. K. , Dutt P. , Suri K. A. , and Qazi G. N. , Immunomodulatory Activity of Biopolymeric Fraction RLJ-NE-205 From *Picrorhiza kurroa* , International Immunopharmacology. (2006) 6, no. 10, 1543–1549, 10.1016/j.intimp.2006.05.002, 2-s2.0-33747072115, 16919826. 16919826

[31] Sharma U. , Bala M. , Kumar N. , Singh B. , Munshi R. K. , and Bhalerao S. , Immunomodulatory Active Compounds From *Tinospora cordifolia* , Journal of Ethnopharmacology. (2012) 141, no. 3, 918–926, 10.1016/j.jep.2012.03.027, 2-s2.0-84861333260, 22472109. 22472109

[32] Agarwal R. , Diwanay S. , Patki P. , and Patwardhan B. , Studies on Immunomodulatory Activity of *Withania somnifera* (Ashwagandha) Extracts in Experimental Immune Inflammation, Journal of Ethnopharmacology. (1999) 67, no. 1, 27–35, 10.1016/S0378-8741(99)00065-3, 2-s2.0-0032788630, 10616957. 10616957

[33] Aftab T. , Ferreira J. F. S. , Khan M. M. A. , and Naeem M. , Artemisia annua-pharmacology and biotechnology, 2014, Springer.

[34] Ivanescu B. , Miron A. , and Corciova A. , Sesquiterpene Lactones from *Artemisia* Genus: Biological Activities and Methods of Analysis, Journal of Analytical Methods in Chemistry. (2015) 2015, no. 1, 247685, 10.1155/2015/247685, 2-s2.0-84944222171. 26495156 PMC4606394

[35] Zhang Y. X. and Sun H. X. , Immunosuppressive Effect of Ethanol Extract of *Artemisia annua* on Specific Antibody and Cellular Responses of Mice Against Ovalbumin, Immunopharmacology and Immunotoxicology. (2009) 31, no. 4, 625–630, 10.3109/08923970902932954, 2-s2.0-70449132576. 19874232

[36] Islamuddin M. , Chouhan G. , Farooque A. , Dwarakanath B. S. , Sahal D. , and Afrin F. , Th1-Biased Immunomodulation and Therapeutic Potential of *Artemisia annua* in Murine Visceral Leishmaniasis, PLoS Neglected Tropical Diseases. (2015) 9, no. 1, e3321, 10.1371/journal.pntd.0003321, 2-s2.0-84922244139, 25568967. 25568967 PMC4287499

[37] Wojtkowiak-Giera A. , Derda M. , Kosik-Bogacka D. , Kolasa-Wołosiuk A. , Solarczyk P. , Cholewiński M. , Wandurska-Nowak E. , Jagodziński P. P. , and Hadaś E. , Influence of *Artemisia annua* L. on Toll-Like Receptor Expression in Brain of Mice Infected With Acanthamoeba Sp, Experimental Parasitology. (2018) 185, 17–22, 10.1016/j.exppara.2018.01.008, 2-s2.0-85042127573, 29317241. 29317241

[38] Wojtkowiak-Giera A. , Derda M. , Kosik-Bogacka D. , Kolasa-Wołosiuk A. , Solarczyk P. , Cholewiński M. , Wandurska-Nowak E. , Jagodziński P. P. , and Hadaś E. , The Modulatory Effect of *Artemisia annua* L. on Toll-Like Receptor Expression in *Acanthamoeba* Infected Mouse Lungs, Experimental Parasitology. (2019) 199, 24–29, 10.1016/j.exppara.2019.02.011, 2-s2.0-85062715653. 30796912

[39] Zhang L. , Reddy N. , Khoo C. S. , and Koyyalamudi S. R. , Structural Characterization and In-Vitro Antioxidant and Immunomodulatory Activities of Polysaccharide Fractions Isolated From *Artemisia annua* L, Molecules. (2022) 27, no. 11, 10.3390/molecules27113643, 35684579. PMC9182033 35684579

[40] Alzohairy M. A. , Therapeutics Role of *Azadirachta indica* (Neem) and Their Active Constituents in Diseases Prevention and Treatment, Evidence-Based Complementary and Alternative Medicine. (2016) 2016, no. 1, 7382506, 10.1155/2016/7382506, 2-s2.0-84962297840. 27034694 PMC4791507

[41] Paul R. , Prasad M. , and Sah N. K. , Anticancer Biology of *Azadirachta indica* L (Neem): A Mini Review, Cancer Biology & Therapy. (2011) 12, no. 6, 467–476, 10.4161/cbt.12.6.16850, 2-s2.0-80052859163, 21743298. 21743298

[42] Morris J. , Gonzales C. B. , De La Chapa J. J. , Cabang A. B. , Fountzilas C. , Patel M. , Orozco S. , and Wargovich M. J. , The Highly Pure Neem Leaf Extract, SCNE, Inhibits Tumorigenesis in Oral Squamous Cell Carcinoma via Disruption of Pro-Tumor Inflammatory Cytokines and Cell Signaling, Frontiers in Oncology. (2019) 9, no. 9, 10.3389/fonc.2019.00890, 2-s2.0-85072842691. PMC6753233 31572681

[43] Nitesh K. and Sunil K. S. , Ethnopharmacological Properties of *Curcuma longa* , International Journal of Pharmaceutical Science and Research. (2013) 4, no. 1, 103–112, 10.13040/IJPSR.0975-8232.4(1).03-12.

[44] Murielle M. and Batra S. K. , Potential Applications of Curcumin and Its Novel Synthetic Analogs and Nanotechnology-Based Formulations in Cancer Prevention and Therapy, Chinese Medicine. (2011) 6, no. 1, 10.1186/1749-8546-6-31, 2-s2.0-80052028983. PMC3177878 21859497

[45] Bimonte S. , Barbieri A. , Palma G. , Luciano A. , Rea D. , and Arra C. , Curcumin Inhibits Tumor Growth and Angiogenesis in an Orthotopic Mouse Model of Human Pancreatic Cancer, BioMed Research International. (2013) 2013, 810423, 10.1155/2013/810423, 2-s2.0-84890082601. 24324975 PMC3842048

[46] Lee G. H. , Lee H. Y. , Choi M. K. , Chung H. W. , Kim S. W. , and Chae H. J. , Protective Effect of *Curcuma longa* L. Extract on CCl_4_ Induced Acute Hepatic Stress, BMC Research Notes. (2017) 10, no. 1, 10.1186/s13104-017-2409-z, 2-s2.0-85010951240. PMC5286822 28143589

[47] Momtazi-Borojeni A. A. , Mohammadian Haftcheshmeh S. , Esmaeili S. A. , Johnston T. P. , Abdollahi E. , and Sahebkar A. , Curcumin: A Natural Modulator of Immune Cells in Systemic Lupus Erythematosus, Autoimmunity Reviews. (2018) 17, no. 2, 125–135, 10.1016/j.autrev.2017.11.016, 2-s2.0-85037038580, 29180127. 29180127

[48] Abdollahi E. , Momtazi A. A. , Johnston T. P. , and Sahebkar A. , Therapeutic Effects of Curcumin in Inflammatory and Immune-Mediated Diseases: A Nature-Made Jack-of-All-Trades?, Journal of Cellular Physiology. (2018) 233, no. 2, 830–848, 10.1002/jcp.25778, 2-s2.0-85012276107, 28059453. 28059453

[49] Karimian M. S. , Pirro M. , Johnston T. P. , Majeed M. , and Sahebkar A. , Curcumin and Endothelial Function: Evidence and Mechanisms of Protective Effects, Current Pharmaceutical Design. (2017) 23, no. 17, 2462–2473, 10.2174/1381612823666170222122822, 2-s2.0-85017467271, 28228072. 28228072

[50] Zhang B. , Swamy S. , Balijepalli S. , Panicker S. , Mooliyil J. , Sherman M. A. , Parkkinen J. , Raghavendran K. , and Suresh M. V. , Direct Pulmonary Delivery of Solubilized Curcumin Reduces Severity of Lethal Pneumonia, FASEB Journal. (2019) 33, no. 12, 13294–13309, 10.1096/fj.201901047RR, 31530014. 31530014 PMC6894047

[51] Arora P. , Li W. , Huang X. , Yu W. , Huang R. , Jiang Q. , and Chen C. , Metabolic Reconfiguration Activates Stemness and Immunomodulation of PDLSCs, International Journal of Molecular Sciences. (2022) 23, no. 7, 10.3390/ijms23074038. PMC8999739 35409397

[52] Rahimi K. , Ahmadi A. , Hassanzadeh K. , Soleimani Z. , Sathyapalan T. , Mohammadi A. , and Sahebkar A. , Targeting the Balance of T Helper Cell Responses by Curcumin in Inflammatory and Autoimmune States, Autoimmunity Reviews. (2019) 18, no. 7, 738–748, 10.1016/j.autrev.2019.05.012, 2-s2.0-85065082789, 31059845. 31059845

[53] Shafabakhsh R. , Pourhanifeh M. H. , Mirzaei H. R. , Sahebkar A. , Asemi Z. , and Mirzaei H. , Targeting Regulatory T Cells by Curcumin: A Potential for Cancer Immunotherapy, Pharmcological Research. (2019) 147, 104353, 10.1016/j.phrs.2019.104353, 2-s2.0-85068968700. 31306775

[54] Soni D. and Grover A. , Picrosides From *Picrorhiza kurroa* as Potential Anticarcinogenic Agents, Biomedicine and Pharmacotherapy. (2019) 109, no. 3, 1680–1687, 10.1016/j.biopha.2018.11.048, 2-s2.0-85056759388, 30551422. 30551422

[55] Gupta R. , Vijay K. B. , Manas M. , Bajaj V. K. , Katariya P. , and Yadav S. , Evaluation of Antidiabetic and Antioxidant Activity of *Moringa oleifera* in Experimental Diabetes, Journal of Diabetes. (2012) 4, no. 2, 164–171, 10.1111/j.1753-0407.2011.00173.x, 2-s2.0-84861170421. 22103446

[56] Kumar R. , Gupta Y. K. , Singh S. , and Raj A. , Anti-Inflammatory Effect of *Picrorhiza kurroa* in Experimental Models of Inflammation, Planta Medica. (2016) 82, no. 16, 1403–1409. 27163229 10.1055/s-0042-106304

[57] Joy K. L. , Rajesh Kumar N. V. , Kuttan G. , and Kuttan R. , Effect of *Picrorrhiza kurroa* Extract on Transplanted Tumours and Chemical Carcinogenesis in Mice, Journal of Ethnopharmacology. (2000) 71, no. 1-2, 261–266, 10.1016/S0378-8741(00)00168-9, 2-s2.0-0033935159. 10904172

[58] Dangi S. Y. , Jolly C. I. , and Narayanan S. , Antihypertensive Activity of the Total Alkaloids From the Leaves of Moringa Oleifera, Pharmceutical Biology. (2002) 40, no. 2, 144–148, 10.1076/phbi.40.2.144.5847, 2-s2.0-0036064837.

[59] Gbankoto A. , Sindete M. , Adjagba M. , Sangare M. , Attakpa E. , and Awede B. , Antihypertensive Effects of *Moringa oleifera* Leaf Extract Lam. (Moringaceae) in NG-nitro-L-arginine-methyl ester-induced Hypertensive Rats, National Journal of Physiology, Pharmacy and Pharmacology. (2019) 9, no. 12, 1257–1266, 10.5455/njppp.2019.9.1034231102019.

[60] Laili R. D. , Martati E. , and Rifa I. M. , Immunomodulator Effect of *Moringa oleifera* Leaves Fermented by *Lactobacillus plantarum* FNCC 0137 on *Salmonella typhi* infected Balb/C Mice, Research Journal of Pharmacy and Technology. (2019) 12, no. 8, 3595–3601, 10.5958/0974-360X.2019.00613.9.

[61] Dong Z. , Li C. , Huang Q. , Zhang B. , Fu X. , and Liu R. H. , Characterization of a Novel Polysaccharide From the Leaves of *Moringa oleifera* and Its Immunostimulatory Activity, Journal of Functional Foods. (2018) 49, 391–400, 10.1016/j.jff.2018.09.002, 2-s2.0-85053063182.

[62] He T. B. , Huang Y. P. , Huang Y. , Wang X. J. , Hu J. M. , and Sheng J. , Structural Elucidation and Antioxidant Activity of an Arabinogalactan From the Leaves of *Moringa oleifera* , International Journal of Biologivcal Macromolecules. (2018) 112, 126–133, 10.1016/j.ijbiomac.2018.01.110, 2-s2.0-85041553337, 29366898. 29366898

[63] Kuddus M. , Ginawi A. M. , and Al-Hazimi A. , Cannabis Sativa: An Ancient Wild Edible Plant of India, Emirates Journal of Food and Agriculture. (2013) 25, no. 10, 736–745, 10.9755/ejfa.v25i10.16400, 2-s2.0-84886550544.

[64] Hampson A. J. , Grimaldi M. , Lolic M. , Wink D. , Rosenthal R. , and Axelrod J. , Neuroprotective Antioxidants From Marijuanaa, Annals of the New York Academy of Sciences. (2000) 899, no. 1, 274–282, 10.1111/j.1749-6632.2000.tb06193.x. 10863546

[65] Amalraj A. , Pius A. S. , Gopi S. , and Gopi S. , Biological Activities of Curcuminoids, Other Biomolecules From Turmeric and Their Derivatives—A Review, Jorunal of Traditional and Complementary Medicines. (2017) 7, no. 2, 205–233, 10.1016/j.jtcme.2016.05.005, 2-s2.0-84989351013. PMC5388087 28417091

[66] Lal S. , Shekher A. , Narula A. S. , Abrahamse H. , and Gupta S. C. , Cannabis and Its Constituents for Cancer: History, Biogenesis, Chemistry and Pharmacological Activities, Pharmacological Research. (2021) 163, 10.1016/j.phrs.2020.105302. 33246167

[67] Makare N. , Bodhankar S. , and Rangari V. , Immunomodulatory Activity of Alcoholic Extract of *Mangifera indica* L. in Mice, Jorunal of Ethnopharmacology. (2001) 78, no. 2-3, 133–137, 10.1016/S0378-8741(01)00326-9, 2-s2.0-0034776246, 11694357. 11694357

[68] Ediriweera M. K. , Tennekoon K. H. , and Samarakoon S. R. , A Review on Ethnopharmacological Applications, Pharmacological Activities, and Bioactive Compounds of *Mangifera indica* (Mango), Evidence-Based Complementary and Alternative Medicine. (2017) 2017, no. 1, 6949835, 10.1155/2017/6949835, 2-s2.0-85042633347. 29456572 PMC5804368

[69] Kumolosasi E. , Ibrahim S. N. , Shukri S. M. , and Ahmad W. , Immunostimulant Activity of Standardised Extracts of *Mangifera indica* Leaf and *Curcuma domestica* Rhizome in Mice, Tropical Journal of Pharmceutical Research. (2018) 17, no. 1, 77–84, 10.4314/tjpr.v17i1.12, 2-s2.0-85041584446.

[70] Ishida M. , Sasaki T. , Nishi K. , Tamamoto T. , and Sugahara T. , Suppressive Effect of Ethanol Extract From Mango (*Mangifera indica* L.) Peel on IgE Production In Vitro and In Vivo, Bioscience, Biotechnology and Biochemistry. (2018) 82, no. 4, 732–739, 10.1080/09168451.2017.1412250, 2-s2.0-85044995225, 29297259. 29297259

[71] Gupta G. and Rana A. , PHCOG MAG.: Plant Review *Withania somnifera* , Pharmacognosy Reviews. (2007) 1, no. 1, 129–136.

[72] Davis L. and Kuttan G. , Immunomodulatory Activity of *Withania somnifera* , Journal of Ethnopharmacology. (2000) 71, no. 1-2, 193–200, 10.1016/S0378-8741(99)00206-8, 2-s2.0-0033935505. 10904163

[73] Machiah D. K. , Girish K. S. , and Gowda T. V. , A Glycoprotein From a Folk Medicinal Plant, *Withania somnifera*, Inhibits Hyaluronidase Activity of Snake Venoms, Comparative Biochemistry and Physiology Part C: Toxicology and Pharmacology. (2006) 143, no. 2, 158–161, 10.1016/j.cbpc.2006.01.006, 2-s2.0-33646167874, 16513428. 16513428

[74] Kaur T. , Singh H. , Mishra R. , Manchanda S. , Gupta M. , Saini V. , Sharma A. , and Kaur G. , *Withania somnifera* as a Potential Anxiolytic and Immunomodulatory Agent in Acute Sleep Deprived Female Wistar Rats, Molecular and Cellular Biochemistry. (2017) 427, no. 1-2, 91–101, 10.1007/s11010-016-2900-1, 2-s2.0-85006856512, 28004351. 28004351

[75] Farahnejad Z. , Ghazanfari T. , and Yaraee R. , Immunomodulatory Effects of *Aloe vera* and Its Fractions on Response of Macrophages Against *Candida albicans* , Immunopharmacology and Immunotoxicology. (2011) 33, no. 4, 676–681, 10.3109/08923973.2011.560158, 2-s2.0-80755181476, 21401385. 21401385

[76] Aranda-Cuevas B. , Tamayo-Cortez J. , Vargas L. V. , Islas-Flores I. , Arana-Argáez V. , Solís-Pereira S. , Cuevas-Glory L. , and Méndez C. H. , Assessment of the Immunomodulatory Effect of Aloe vera Polysaccharides Extracts on Macrophages Functions, Emirates Journal of Food and Agriculture. (2020) 32, no. 6, 408–416, 10.9755/ejfa.2020.v32.i6.2101.

[77] Dziewulska D. , Stenzel T. , Śmiałek M. , Tykałowski B. , and Koncicki A. , The Impact of *Aloe vera* and Licorice Extracts on Selected Mechanisms of Humoral and Cell-Mediated Immunity in Pigeons Experimentally Infected With PPMV-1, BMC Veterinary Research. (2018) 14, no. 1, 10.1186/s12917-018-1467-3, 2-s2.0-85046259676. PMC5930501 29716604

[78] Hasan N. , Ahmad N. , Zohrameena S. , Khalid M. , and Akhtar J. , Asparagus Racemosus: For Medicinal Uses and Pharmacological Actions, International Journal of Advanced Research. (2016) 4, no. 3, 259–267.

[79] Gautam M. , Saha S. , Bani S. , Kaul A. , Mishra S. , Patil D. , and Patwardhan B. , Immunomodulatory Activity of *Asparagus racemosus* on Systemic Th1/Th2 Immunity: Implications for Immunoadjuvant Potential, Journal of Ethnopharmacology. (2009) 121, no. 2, 241–247, 10.1016/j.jep.2008.10.028, 2-s2.0-57949107702. 19038322

[80] Pise M. V. , Rudra J. A. , Upadhyay A. , Rudra J.,. A. , and Upadhyay A. , Immunomodulatory Potential of Shatavarins Produced From *Asparagus racemosus* Tissue Cultures, Journal of Natural Science, Biology and Medicine. (2015) 6, no. 2, 415–420, 10.4103/0976-9668.160025, 2-s2.0-84937030053, 26283842. 26283842 PMC4518422

[81] Aranha I. and Venkatesh Y. P. , Humoral Immune and Adjuvant Responses of Mucosally-Administered *Tinospora cordifolia* Immunomodulatory Protein in BALB/c Mice, Journal of Ayurveda and Integrative Medicine. (2020) 11, no. 2, 140–146, 10.1016/j.jaim.2017.10.006, 2-s2.0-85056622198. 30455069 PMC7329723

[82] Kaushik A. , Husain A. , Awasthi H. , Singh D. , Khan R. , and Mani D. , Antioxidant and Hepatoprotective Potential of Swaras and Hima Extracts of *Tinospora cordifolia* and *Boerhavia diffusa* in Swiss Albino Mice, Pharmacognosy Magazine. (2017) 13, no. supplement 3, S658–S662, 10.4103/pm.pm_448_16, 2-s2.0-85032939846. 29142429 PMC5669112

[83] Sharif A. A. , Unyah N. Z. , Nordin N. , Basir R. , Wana M. N. , Alapid Ahmad A. , Mustapha T. , and Majid R. A. , Susceptibility of *Toxoplasma gondii* to Ethanolic Extract of *Tinospora crispain* Vero Cells, Evidence-Based Complement Alternative Medicine. (2019) 2019, 2916547, 10.1155/2019/2916547. PMC6885813 31827548

[84] Lee D. C. , Yang C. L. , Chik S. C. , Li J. C. , Rong J. H. , Chan G. C. , and Lau A. S. , Bioactivity Guided Identification and Cell Signaling Technology to Delineate the Immunomodulatory Effects of *Panax ginseng* on Human Promonocytic U937 Cells, Journal of Translational Medicine. (2009) 7, no. 1, 10.1186/1479-5876-7-34, 2-s2.0-66949121846. PMC2689162 19442267

[85] Wang L. , Huang Y. , Yin G. , Wang J. , Wang P. , Chen Z. Y. , and Ren G. , Antimicrobial Activities of Asian Ginseng, American Ginseng, and Notoginseng, Phytotherapy Research. (2020) 34, no. 6, 1126–1236, 10.1002/ptr.6605. 31885119

[86] Kim J. , Byeon H. , and Min H. , Effects of Ginsenosides on Regulatory T Cell Differentiation, Food Science and Biotechnology. (2018) 27, no. 1, 227–232, 10.1007/s10068-017-0255-3, 2-s2.0-85041445457, 30263744. 30263744 PMC6049740

[87] Nguyen C. T. and Rhee D. K. , Panax Ginseng as a Potential Modulator of Macrophages, Macrophage. (2016) 3, e1082, 10.14800/Macrophage.1082.

[88] Sapna B. and Nancy P. , Phytochemical Analysis and Chromatographic Evaluation of Alcoholic Extract of *Dillenia indica* Linn. Leaves, International Journal of Pharmaceutical Science and Research. (2015) 6, no. 7, 2799–2812.

[89] Bagherwal P. , Dahake A. P. , and Chakma C. , Immunostimulant Activity of Aqueous Extract Roots of *Glycyrrhiza glabra* , Research Journal of Pharmacology and Pharmadyanmics. (2009) 1, no. 3, 120–124.

[90] Richard S. A. , Exploring the Pivotal Immunomodulatory and Anti-Inflammatory Potentials of Glycyrrhizic and Glycyrrhetinic Acids, Mediators of Inflammation. (2021) 2021, no. 1, 6699560, 10.1155/2021/6699560, 33505216. 33505216 PMC7808814

[91] Qiong H. , Han L. , Zhang N. , Chen H. , Yan K. , Zhang Z. , Ma Y. , and Xu J. , Glycyrrhizin Improves the Pathogenesis of Psoriasis Partially Through IL-17A and the SIRT1-STAT3 Axis, BMC Immunology. (2021) 22, no. 1, 10.1186/s12865-021-00421-z. PMC8161965 34044769

[92] Madhuri S. , Govind P. , and Sharma V. K. , Antioxidant, Immunomodulatory and Anticancer Activities of *Emblica officinalis*: An Overview, International Research Journal of Pharmacy. (2011) 2, no. 8, 38–42.

[93] Zhao T. , Sun Q. , Marques M. , and Witcher M. , Anticancer Properties of *Phyllanthus emblica* (Indian Gooseberry), Oxidative Medicine and Cellular Longevity. (2015) 2015, no. 1, 950890, 10.1155/2015/950890, 2-s2.0-84935037959. 26180601 PMC4477227

[94] Singh M. K. , Yadav S. S. , Gupta V. , and Khattri S. , Immunomodulatory Role of *Emblica officinalis* in Arsenic Induced Oxidative Damage and Apoptosis in Thymocytes of Mice, BMC Complementary and Alternative Medicine. (2013) 13, no. 1, 10.1186/1472-6882-13-193, 2-s2.0-84880954801. PMC3733846 23889914

[95] Kumar A. , Singh A. , and Singh B. , Assessment of Therapeutic Potential of *Phyllanthus emblica* (Amla): A Natural Godsend, International Journal of Cell Science and Biotechnology. (2014) 3, 4–14.

[96] Bharati B. D. , Sharma P. K. , Kumar N. , Dudhe R. , and Bansal V. , Pharmacological Activity of *Andrographis paniculata*: A Brief Review, Pharmacologyonline. (2011) 1, 1–10.

[97] Hossain M. S. , Urbi Z. , Sule A. , and Rahman K. M. , *Andrographis paniculata* (Burm. F.) Wall. Ex Nees: A Review of Ethnobotany, Phytochemistry, and Pharmacology, The Scientific World Journal. (2014) 2014, no. 1, 274905, 10.1155/2014/274905, 2-s2.0-84921478850. 25950015 PMC4408759

[98] Kandanur S. G. S. , Tamang N. , Golakoti N. R. , and Nanduri S. , Andrographolide: A Natural Product Template for the Generation of Structurally and Biologically Diverse Diterpenes, European Journal of Medicinal Chemistry. (2019) 176, 513–533, 10.1016/j.ejmech.2019.05.022, 2-s2.0-85066265349, 31151068. 31151068

[99] Rajanna M. , Bharathi B. , Shivakumar V. , Deepak M. , Prabakaran D. , Vijayabhaskar T. , and Arun B. , Immunomodulatory Effects of *Andrographis paniculata* Extract in Healthy Adults–An Open-Label Study, Journal of Ayurveda and Integrated Medicines. (2021) 12, no. 3, 529–534, 10.1016/j.jaim.2021.06.004, 34376353. PMC8377179 34376353

[100] Singh P. , Bhat S. S. , Punnapuzha A. , Bhagavatula A. , Venkanna B. U. , Mohamed R. , and Rao R. P. , Effect of Key Phytochemicals From *Andrographis paniculata*, *Tinospora cordifolia* and *Ocimum sanctum* on PLpro-ISG15 De-Conjugation Machinery—A Computational Approach, Computation. (2022) 10, no. 7, 109–115, 10.3390/computation10070109.

[101] Prakash P. and Gupta N. , Therapeutic Uses of *Ocimum sanctum* Linn (Tulsi) With a Note on Eugenol and Its Pharmacological Actions: A Short Review, Indian Journal of Physiology and Pharmacology. (2005) 49, no. 2, 125–131, 16170979. 16170979

[102] Pattanayak P. , Behera P. , Das D. , and Panda S. , *Ocimum sanctum* Linn. A Reservoir Plant for Therapeutic Applications: An Overview, Pharmacognosy Reviews. (2010) 4, no. 7, 95–105, 10.4103/0973-7847.65323, 2-s2.0-77955701783. 22228948 PMC3249909

[103] Vats V. , Yadav S. P. , and Grover J. K. , Ethanolic Extract of *Ocimum sanctum* Leaves Partially Attenuates Streptozotocin-Induced Alterations in Glycogen Content and Carbohydrate Metabolism in Rats, Journal of Ethnopharmacology. (2004) 90, no. 1, 155–160, 10.1016/j.jep.2003.09.034, 2-s2.0-0347123324. 14698524

[104] Baliga M. S. , Jimmy R. , Thilakchand K. R. , Sunitha V. , Bhat N. R. , Saldanha E. , and Palatty P. L. , *Ocimum sanctum* L (Holy Basil or Tulsi) and Its Phytochemicals in the Prevention and Treatment of Cancer, Nutrition and Cancer. (2013) 65, no. supplement 1, 26–35, 10.1080/01635581.2013.785010, 2-s2.0-84878598430, 23682780. 23682780

[105] Bhalla G. , Kaur S. , Kaur J. , Kaur R. , and Raina P. , Antileishmanial and Immunomodulatory Potential of *Ocimum sanctum* Linn. and *Cocos nucifera* Linn. In Murine Visceral Leishmaniasis, Journal of Parasitic Diseases. (2017) 41, no. 1, 76–85, 10.1007/s12639-016-0753-x, 2-s2.0-84962163481, 28316391. 28316391 PMC5339176

[106] Shafi T. A. , Bansal B. K. , Gupta D. K. , and Nayyar S. , Evaluation of Immunotherapeutic Potential of Ocimum sanctumin Bovine Subclinical Mastitis, Turkish Journal of Veterinary & Animal Sciences. (2016) 40, no. 3, 352–358, 10.3906/vet-1506-96, 2-s2.0-84965050376.

[107] Zhai Z. , Liu Y. , Wu L. , Senchina D. S. , Wurtele E. S. , Murphy P. A. , and Cunnick J. E. , Enhancement of Innate and Adaptive Immune Functions by Multiple *Echinacea* Species, Journal of Medicinal Food. (2007) 10, no. 3, 423–434, 10.1089/jmf.2006.257, 2-s2.0-34848842172. 17887935 PMC2362099

[108] Wang C. Y. , Chiao M. T. , Yen P. J. , Huang W. C. , Hou C. C. , Chien S. C. , Yeh K. C. , Yang W. C. , Shyur L. F. , and Yang N. S. , Modulatory Effects of *Echinacea purpurea* Extracts on Human Dendritic Cells: A Cell- and Gene-Based Study, Genomics. (2006) 88, no. 6, 801–808, 10.1016/j.ygeno.2006.08.011, 2-s2.0-33750957816, 17011161. 17011161

[109] Fu A. , Wang Y. , Wu Y. , Chen H. , Zheng S. , Li Y. , Xu X. , and Li W. , *Echinacea purpurea* Extract Polarizes M1 Macrophages in Murine Bone Marrow-Derived Macrophages Through the Activation of JNK, Journal of Cellular Biochemistry. (2017) 118, no. 9, 2664–2671, 10.1002/jcb.25875, 2-s2.0-85019450143. 28067413

[110] Yang Q. H. , Yang J. , Liu G. Z. , Wang L. , Zhu T. C. , Gao H. L. , and Kou X. G. , Study on In Vitro Anti-Tumor Activity of *Bidens bipinnata* L. Extract, African Journal of Traditional, Complementary and Alternative Medicines. (2013) 10, no. 3, 543–549, 10.4314/ajtcam.v10i3.24, 24146487. PMC3777599 24146487

[111] Chang S. , Chiang Y. , Chang C. , Yeh H. , Shyur L. , Kuo Y. , Wu T. K. , and Yang W. C. , Flavonoids, Centaurein and Centaureidin, From *Bidens pilosa*, Stimulate IFN-*γ* Expression, Journal of Ethnopharmacology. (2007) 112, no. 2, 232–236, 10.1016/j.jep.2007.03.001, 2-s2.0-34248545485, 17408892. 17408892

[112] Gomes A. , Datta P. , Sarkar A. , Dasgupta S. C. , and Gomes A. , Black Tea (*Camellia sinensis*) Extract as an Immunomodulator Against Immunocompetent and Immunodeficient Experimental Rodents, Oriental Pharmacy and Experimental Medicine. (2014) 14, no. 1, 37–45, 10.1007/s13596-013-0134-2, 2-s2.0-84896071875.

[113] Rahayu R. P. , Prasetyo R. A. , Purwanto D. A. , Kresnoadi U. , Iskandar R. P. , and Rubianto M. , The Immunomodulatory Effect of Green Tea (*Camellia sinensis*) Leaves Extract on Immunocompromised Wistar Rats Infected by *Candida albicans* , Veterinary World. (2018) 11, no. 6, 765–770, 10.14202/vetworld.2018.765-770, 2-s2.0-85048438821, 30034167. 30034167 PMC6048092

[114] Chen F. C. , Shen K. P. , Ke L. Y. , Lin H. L. , Wu C. C. , and Shaw S. Y. , Flavonoids From *Camellia sinensis* (L.) O. Kuntze Seed Ameliorates TNF-*α* Induced Insulin Resistance in HepG2 Cells, Saudi Pharmaceutical Journal. (2019) 27, no. 4, 507–516, 10.1016/j.jsps.2019.01.014, 2-s2.0-85060913685, 31061619. 31061619 PMC6488808

[115] Jia L. Y. , Wu X. J. , Gao Y. , Rankin G. O. , Pigliacampi A. , Bucur H. , and Chen Y. C. , Inhibitory Effects of Total Triterpenoid Saponins Isolated From the Seeds of the Tea Plant (*Camellia sinensis*) on Human Ovarian Cancer Cells, Molecules. (2017) 22, no. 10, 10.3390/molecules22101649, 2-s2.0-85032629955, 28974006. PMC6151552 28974006

[116] Harun N. H. , Septama A. W. , Wan Ahmad W. A. N. , and Suppian R. , The Potential of *Centella asiatica* (Linn.) Urban as an Anti-Microbial and Immunomodulator Agent: A Review, Natural Product Sciences. (2019) 25, no. 2, 92–102, 10.20307/nps.2019.25.2.92, 2-s2.0-85071413778.

[117] Zhang A. , Ba X. , Weng X. , Zhao B. , Wang D. , Cao H. , and Huang J. , Immunological Activities of the Aqueous Extracts of *Cistanche deserticola* as a Polysaccharide Adjuvant for Inactivated Foot-and-Mouth Disease Vaccines, Food and Agriculture Immunology. (2021) 32, no. 1, 126–142, 10.1080/09540105.2021.1880551.

[118] Zhang A. , Yang X. , Li Q. , Yang Y. , Zhao G. , Wang B. , and Wu D. , Immunostimulatory Activity of Water-Extractable Polysaccharides From *Cistanche deserticola* as a Plant Adjuvant In Vitro and In Vivo, PLoS One. (2018) 13, no. 1, e0191356, 10.1371/journal.pone.0191356, 2-s2.0-85040985081, 29360858. 29360858 PMC5779666

[119] Feng S. , Yang X. , Weng X. , Wang B. , and Zhang A. , Aqueous Extracts From Cultivated *Cistanche deserticola* Y.C. Ma as Polysaccharide Adjuvant Promote Immune Responses via Facilitating Dendritic Cell Activation, Journaal of Ethnopharmacology. (2021) 277, 114256, 10.1016/j.jep.2021.114256, 34062250. 34062250

[120] Kwan Y. P. , Saito T. , Ibrahim D. , Al-Hassan F. M. S. , Ein O. C. , Chen Y. , and Sasidharan S. , Evaluation of the Cytotoxicity, Cell-Cycle Arrest, and Apoptotic Induction by *Euphorbia hirta* in MCF-7 Breast Cancer Cells, Pharmceutical Biology. (2016) 54, no. 7, 1223–1236. 10.3109/13880209.2015.1064451 26154521

[121] Mojaverrostami S. , Bojnordi M. N. , Ghasemi-Kasman M. , Ebrahimzadeh M. A. , and Hamidabadi H. G. , A Review of Herbal Therapy in Multiple Sclerosis, Advanced Pharmaceutical Bulletin. (2018) 8, no. 4, 575–590, 10.15171/apb.2018.066, 2-s2.0-85057728103. 30607330 PMC6311642

[122] Mu X. and Wang C. , Artemisinins—A Promising New Treatment for Systemic Lupus Erythematosus: A Descriptive Review, Current Rheumatology Reports. (2018) 20, no. 9, 10.1007/s11926-018-0764-y, 2-s2.0-85051108211. 30056574

[123] Van Der Fits L. , Mourits S. , Voerman J. S. , Kant M. , Boon L. , Laman J. D. , and Lubberts E. , Imiquimod-Induced Psoriasis-Like Skin Inflammation in Mice Is Mediated via the IL-23/IL-17 Axis, Journal of Immunology. (2009) 182, no. 9, 5836–5845, 10.4049/jimmunol.0802999, 2-s2.0-66949162673, 19380832. 19380832

[124] Mahdi H. J. , Khan N. A. K. , Asmawi M. Z. B. , Mahmud R. , Vikneswaran A. , and Murugaiyah L. , In Vivo Anti-Arthritic and Anti-Nociceptive Effects of Ethanol Extract of *Moringa oleifera* Leaves on Complete Freund′s Adjuvant (CFA)-Induced Arthritis in Rats, Integrative Medicine Research. (2018) 7, no. 1, 85–94, 10.1016/j.imr.2017.11.002, 29629295. 29629295 PMC5884001

[125] Bharti A. C. , Gupta N. , and Sharma P. K. , Clinical Relevance of Curcumin-Induced Immunosuppression in Living-Related Donor Renal Transplant: An In Vitro Analysis, Experimental and Clinical Transplantation. (2010) 8, no. 2, 161–171. 20565374

